# Multi-Omics Marker Analysis Enables Early Prediction of Breast Tumor Progression

**DOI:** 10.3389/fgene.2021.670749

**Published:** 2021-06-03

**Authors:** Haifeng Xu, Tonje Lien, Helga Bergholtz, Thomas Fleischer, Lounes Djerroudi, Anne Vincent-Salomon, Therese Sørlie, Tero Aittokallio

**Affiliations:** ^1^Department of Cancer Genetics, Institute for Cancer Research, Oslo University Hospital, Oslo, Norway; ^2^Oslo Centre for Biostatistics and Epidemiology (OCBE), University of Oslo, Oslo, Norway; ^3^Institut Curie, Ensemble Hospitalier, Pôle de Médecine Diagnostique et Théranostique, Département de Pathologie, Paris, France; ^4^Institute for Molecular Medicine Finland (FIMM), HiLIFE, University of Helsinki, Helsinki, Finland

**Keywords:** risk signature, breast cancer, disease progression, early detection, machine learning

## Abstract

Ductal carcinoma *in situ* (DCIS) is a preinvasive form of breast cancer with a highly variable potential of becoming invasive and affecting mortality of the patients. Due to the lack of accurate markers of disease progression, many women with detected DCIS are currently overtreated. To distinguish those DCIS cases who are likely to require therapy from those who should be left untreated, there is a need for robust and predictive biomarkers extracted from molecular or genetic profiles. We developed a supervised machine learning approach that implements multi-omics feature selection and model regularization for the identification of biomarker combinations that could be used to distinguish low-risk DCIS lesions from those with a higher likelihood of progression. To investigate the genetic heterogeneity of disease progression, we applied this approach to 40 pure DCIS and 259 invasive breast cancer (IBC) samples profiled with genome-wide transcriptomics, DNA methylation, and DNA copy number variation. Feature selection using the multi-omics Lasso-regularized algorithm identified both known genes involved in breast cancer development, as well as novel markers for early detection. Even though the gene expression-based model features led to the highest classification accuracy alone, methylation data provided a complementary source of features and improved especially the sensitivity of correctly classifying DCIS cases. We also identified a number of repeatedly misclassified DCIS cases when using either the expression or methylation markers. A small panel of 10 gene markers was able to distinguish DCIS and IBC cases with high accuracy in nested cross-validation (AU-ROC = 0.99). The marker panel was not specific to any of the established breast cancer subtypes, suggesting that the 10-gene signature may provide a subtype-agnostic and cost-effective approach for breast cancer detection and patient stratification. We further confirmed high accuracy of the 10-gene signature in an external validation cohort (AU-ROC = 0.95), profiled using distinct transcriptomic assay, hence demonstrating robustness of the risk signature.

## Introduction

Ductal carcinoma *in situ* (DCIS) is a non-invasive precursor to invasive breast cancer (IBC) with low risk of progression (Cowell et al., 2013). Recent advances in breast cancer screening have resulted in an increasing number of women with detected DCIS lesions (Virnig et al., 2010; Seely and Alhassan, 2018; van Seijen et al., 2019), many of which actually will never progress to invasive disease (Page et al., 1982, 1995; Nielsen et al., 1984; Collins et al., 2005; Sanders et al., 2005). To distinguish the DCIS lesions with invasive potential from those that may be left untreated, there is need for robust biomarkers (or risk signatures) for accurate classification between high-risk and low-risk DCIS cases. However, DCIS lesions exhibit heterogeneous clinical, histopathological, and molecular characteristics that may vary considerably between the lesions and as a function of time (Vincent-Salomon et al., 2008). Furthermore, the underlying mechanisms of progression from DCIS to IBC are still poorly understood. The diagnostic classification has therefore considerable uncertainty, and the DCIS lesions may vary from indolent lesions to tumors on the verge of becoming invasive (Gorringe and Fox, 2017). Due to this uncertainty, treatment for DCIS is often extensive, resulting in substantial overtreatment (Esserman et al., 2014; Groen et al., 2017).

Even though histological grade and growth pattern provide some information on disease risk, there is a need for more precise risk prediction methods (Wang et al., 2011; Wallis et al., 2012; Onega et al., 2017). It has been shown that the “intrinsic” breast cancer subtypes (luminal A, luminal B, HER2-enriched, and basal-like) have prognostic significance, and a supervised risk predictor was developed based on the intrinsic subtypes and clinical information (Parker et al., 2009). We have also previously performed comparative analyses across the breast cancer subtypes and identified molecular differences between DCIS and IBC for subtype-specific disease progression (Bergholtz et al., 2020). In these subtype-stratified analyses, prominent molecular differences were identified especially for the basal-like DCIS, which was found to be less proliferative and showed a higher degree of differentiation than the basal-like IBC. However, for clinical use of the risk signatures, there is a need for cost-effective and subtype-agnostic biomarker panels that are widely applicable among diagnosed women regardless of their breast cancer subtype or other risk classifications that would require extensive clinical, histopathological, or molecular information.

In this study, we developed a supervised machine learning approach that implements multi-omics feature selection for the identification of biomarker combinations to distinguish DCIS and IBC cases. As a secondary objective, we identified a robust marker panel to identify those DCIS cases that may have a higher risk of progression (i.e., DCIS cases susceptible to invasion). To investigate the molecular, genetic, and epigenetic heterogeneity of disease progression, we applied the regularized approach to 40 DCIS and 259 IBC samples, profiled with genome-wide transcriptomics, DNA methylation, and DNA copy number variation. For economic clinical implementation, we further investigated the effect of the number of model features on the classification accuracy with each omics measurements. In doing so, we identified a minimal risk signature of 10 highly predictive and subtype-agnostic transcriptomic markers, originating from a single omics platform (microarrays), which could be developed as a decision support tool in clinical practice. We further validated our minimal risk signature in an independent validation cohort (with RNA-seq data) and studied how the signature predicted also lesions between DCIS and IBC classes, as well as relapsing DCIS cases.

## Materials and Methods

### Training Material

As a model training data, we used multi-omics molecular and genomic profiles combined from three patient cohorts, Oslo2, Uppsala, and Milan (Muggerud et al., 2010; Fleischer et al., 2014; Lesurf et al., 2016; Aure et al., 2017; Bergholtz et al., 2020). Each patient cohort contains three levels of omics data from gene expression microarrays, DNA methylation, and DNA copy number. Gene expression was measured with Agilent Sureprint G3 Human Gene Expression 8 × 60 K microarrays (G4851A) (Agilent Technologies, Santa Clare, United States), with Low Input Quick Amp Labeling protocol. The DNA methylation was profiled using the Illumina Infinium Human Methylation 450K microarray (Illumina, CA, United States), following the manufacturer’s instructions, and preprocessed with subset quantile normalization (Touleimat and Tost, 2012). The DNA copy number changes were profiled using Affymetrix SNP 6.0 arrays (Affymetrix, Santa Clara, United States) at Aros Applied Biotechnology (Aarhus, Denmark), following the manufacturer’s instructions. In total, there were 370 patients included in these three cohorts. We included only patients with all three omics data levels, resulting in 299 patients as our training material, including 40 DCIS cases and 259 IBC cases (Supplementary Figure 1 and Supplementary Data 1).

The gene expression and DNA copy number changes were mapped to protein-coding genes to make it easier to interpret the results and integrate across the omics data. To investigate the effect of DNA methylation data processing on predictive modeling, we considered two versions of the DNA methylation data. The first option was to use directly the original CpG level methylation data as model features, and therefore we performed feature preselection using only CpGs thought to be involved in important biological variation between breast cancer samples (*N* = 44,263 CpGs) (Fleischer et al., 2017). These CpGs were thought to be involved in one of four breast cancer biological properties, namely, regulation of estrogen signaling, regulation of non-estrogen-related proliferation, fibroblast infiltration, or immune infiltration. The CpGs were located both inside and outside CpG islands and were enriched in both enhancers and promoters. The second option used gene-level processing, where we calculated a methylation score to represent each protein-coding gene using a principal component analysis (PCA), taking into account the variation of all CpGs mapped to a gene, similarly as before (Bergholtz et al., 2020). The second option leads to gene-level features, whereas in the first option, each gene can be associated with hundreds of CpGs.

### Validation Material

The validation data set was collected at Institute Curie, France (referred to as Curie Cohort), where the gene expression was profiled using RNA sequencing with the Illumina HiSeq2500 sequencer. The read counts were normalized with the rlog and cpm options in edgeR (v3.1.2) and DESeq2 (v1.4.5) R-packages, respectively (Robinson et al., 2009; Love et al., 2017). Pseudocount data were calculated as log_1__0_(RNAseq count + 1), and it was centered for each gene around the mean of the pseudocounts. The validation cohort included 18 pure DCIS cases and 20 IBC cases, as well as 16 micro-invasive (MI) DCIS cases, which are DCIS lesions with invasive foci of maximum 1 mm.

### Classification Models

Our main objective was to identify the most discriminating molecular and genetic differences between DCIS and IBC, regardless of their intrinsic subtype and the nuclear grade. We initially constructed Lasso, Support Vector Machine (SVM), and Random Forest (RF) models based on each type of omics data (gene expression, DNA methylation, and DNA copy number). We used the R-package “glmnet” to build Lasso models, R-package “e1071” to build SVM models, and R-package “randomForest” to build RF models (Liaw and Wiener, 2002; Friedman et al., 2010; Meyer et al., 2019). To assess the classification accuracy, we used 10-fold cross validation (CV), where the training dataset was divided into 10-fold, testing on each fold at a time, while the remaining ninefold were used for the model estimation (sub-training set). Stratified CV was used to make sure each CV fold had the same proportion of breast cancer subtypes. To test the generalizability of the Lasso models, and to avoid selection bias, we used nested cross-validation, where another 10-fold CV was applied within each sub-training set to determine the optimal model regularization parameters, e.g., the lambda and beta values of the Lasso model. The other model parameters were set to their default values. When training the SVM models, we used Recursive Feature Elimination (RFE) implemented in the R-package “caret” to select the model features (Kuhn, 2008). The size parameter of RFE was set to a vector (2, 5, 10, and 20), the parameter “number” of the rfeControl function was set to 5, and the kernel parameter was set to svmRadial to use the radial kernel. We used 10-fold CV for the SVM models, and in each fold, RFE was run to select the model features using nested CV.

### Evaluation Metrics

To evaluate the predictive accuracy, we used Area Under the ROC Curve (AU-ROC) and Area under the Precision-Recall Curve (AU-PRC) (Supplementary Figure 2). Moreover, classification cutoff-specific evaluation metrics, such as sensitivity and specificity, were also recorded to evaluate the trade-off between correctly classifying either DCIS or IBC cases. For avoiding overtreatment, it is especially important to correctly predict true DCIS cases, and therefore we labeled DCIS as positive and IBC as negative cases. Accordingly, sensitivity TP/(TP + FN) refers to the rate of how many DCIS cases are correctly classified, while specificity TN/(TN + FP) refers to the percentage of correctly classified IBC cases. Balanced accuracy is defined as the average of sensitivity and specificity. Precision–Recall analysis provides an alternative evaluation metric for the unbalanced classification problem. The AU-ROC and the AU-PRC were plotted and calculated with the R-packages “PRROC” (Grau et al., 2015) and “pROC” (Robin et al., 2011), respectively. As a continuous evaluation metric, we used Mean Squared Error (MSE), where MSE values close to zero indicate more accurate models.

### Multi-Omics Classifiers

To test whether integrating the three types of omics data improved the prediction accuracies, we combined gene expression data, DNA methylation, and DNA copy number data together in a single Lasso model. The CpG-level and gene-level methylation data were combined separately with the other data types to investigate their respective predictive contribution. To unify the scales between the different data types, we applied z-score scaling over each feature (gene or CpG) and then combined the z-scored features into a single model.

### Limiting Model Complexity

To test the effect of limiting the maximum allowed number of model features on the prediction accuracy, we adjusted the parameter “dfmax” of the glmnet function, which limits the maximum number of variables in the Lasso-regularized model (Friedman et al., 2010). We varied the dfmax parameter from 2 to 51 with each separate omics data and their combination using nested CV to explore the most predictive feature subsets and to construct a maximally sparse, cost-effective, and transparent models for economic clinical implementation.

### Robust Gene Selection

We considered the common features identified by the two classification models, SVM and Lasso, as robust biological signatures. To further improve the reliability of these signatures, and to avoid reporting unstable features, we considered only those features that were returned more than five times during the 10-fold CV (i.e., >50% of the folds), where each feature can be selected up to 10 times. This analysis was limited to the gene expression data only (without using z-scoring), since gene expression data was found generally most predictive.

### Model Validation

In the validation phase, we trained a new Lasso model using the subset of 10 most robust genes on the entire training set and tested its predictive power on the validation set (the Curie Cohort). Only RNA-seq transcriptomics data were available in the validation set. We used z-score scaling over each gene separately in the training and validation sets to normalize their scales between the microarray and RNA sequencing data. The model outcome was the predicted class probability in DCIS vs. IBC classification for each validation case separately.

### Existing Risk Scores

We compared the 10-gene signature against three existing risk scores. The first was ROR, risk of recurrence after surgical treatment for IBC, calculated based on expression of the PAM50 genes (Parker et al., 2009). Firstly, the correlation to the four breast cancer subtypes (Basal-like, Her2-enriched, Luminal A, and Luminal B) was calculated, and the ROR score was then defined as a weighted sum of the four correlations. We also calculated an invasiveness score based on a previously proposed 64-gene signature (Anastassiou et al., 2011). We summarized the 64-signature using z-score to obtain an invasiveness score for each sample and then used the mean value of each case as the final invasive score. As the third comparison score, we used the Oncotype DX^®^ DCIS Score that has been suggested to quantify the risk of developing an ipsilateral breast event (i.e., local recurrence of DCIS or invasive carcinoma) (Solin et al., 2013). The original DCIS score was calculated using qPCR expression values from 12 genes. However, since our training cohort included normalized microarray expression data, we did not perform the first step of the DCIS Score calculation, i.e., normalizing seven signature genes relative to the expression of five housekeeping genes. The ROR, invasiveness, and DX^®^ DCIS scores were included in the simple linear model using function “glm” from basic R, where only the score was used when building these models using 10-fold CV.

### Identification of Misclassified DCIS Cases

Some DCIS cases may never progress to IBC and will remain intraductal, while other DCIS lesions may have future invasive potential but were discovered while still intraductal. We hypothesized that even though some lesions are discovered while still intraductal, they may carry molecular or genomic changes that distinguish them from the low-risk DCIS cases that will never progress. To address the secondary questions of whether we can divide DCIS samples into two groups, low- and high-risk DCIS, and how accurately we can find those higher-risk DCIS cases that might carry the potential for future invasion, we built additional machine learning models based on gene expression and DNA methylation data, and the cases incorrectly classified by more than one model-data combinations were considered for further scrutiny. Next, we used so-called pseudo labeling, where the repeatedly misclassified DCIS cases were relabeled as IBC, then retrained a Lasso model with 10-fold nested CV and checked whether or not its classification accuracy increased, compared to the original Lasso model with the original class labeling.

## Results

### Predictive Model Development in Multi-Omics Data

We started by testing various prediction algorithms, including Lasso, SVM, and RF, to classify the patient samples of the training cohort into two groups, DCIS and IBC. These algorithms were evaluated in terms of their classification accuracy and robustness in the heterogeneous omics data (gene expression, DNA copy number, and DNA methylation). In the initial runs, the classifiers were allowed to freely make use of an unlimited number of the omics features (genes and CpGs), and nested CV was then used to evaluate the predictive power of the models and the selected feature panels. In this section, we show the results of the Lasso model that performed generally the best, while the results of RF and SVM models are provided in Supplementary Tables 2, 3, respectively, showing similar performance trends with slightly decreased accuracies.

**FIGURE 1:**
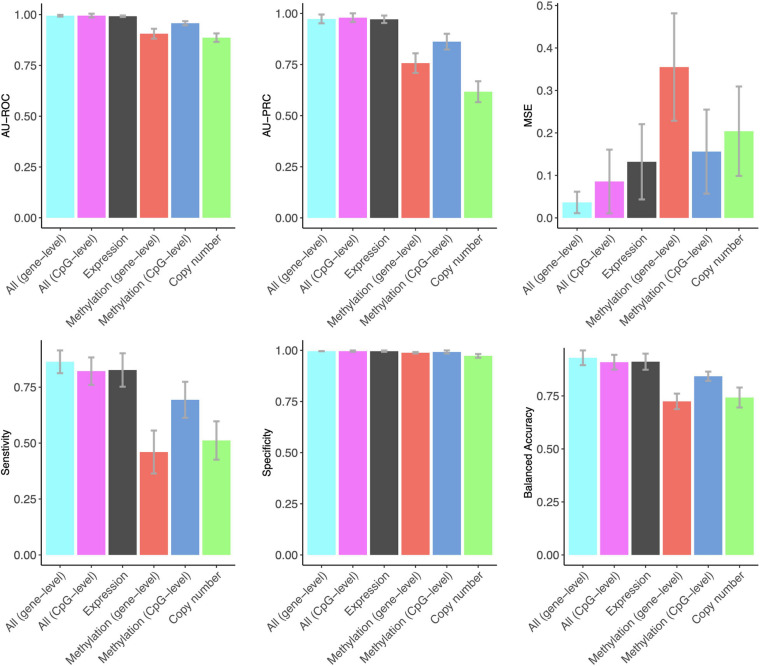
The predictive contribution of omics profiles and their combinations to DCIS vs. IBC classification. The bars show the average accuracy over 10 CV folds, and the error bars indicate the standard error of the mean (SEM). These data correspond to those shown in Supplementary Table 1. ROC and PRCs of CV folds using various omics data combinations are shown in Supplementary Figure 3.

Notably, gene expression features provided the best overall accuracy among the single omics datasets when using summary metrics AU-ROC and AU-PRC (Figure 1). Interestingly, the CpG-level methylation data provided almost as high AU-ROC levels, but the Lasso model selected more than three times the CpG features compared to expression features, and the CpG model had much a lower AU-PRC value (Figure 1). DNA copy number variation profiles showed the poorest performance among the three omics datasets, even though the Lasso model selected the largest number of copy number features, suggesting that copy number changes do not contain a sufficient predictive signal for the classification between DCIS and IBC cases. All the omics profiles resulted in close to perfect specificity (Figure 1).

The combined use of the three omics features in a single Lasso model using z-score scaling resulted in similar AU-ROC and AU-PRC values when using the gene expression features alone (Figure 1). However, the sensitivity of correctly classified DCIS cases increased when using all the omics data together. In clinical practice, sensitivity is more important for avoiding overtreatment. Omics data integration also led to higher levels of balanced accuracy, while the specificity of correctly classifying IBC cases remained perfect, similar to that when using the gene expression data only. The two versions of the DNA methylation data provided a similar contribution to the multi-omics Lasso model; however, the gene-level methylation features led to slightly increased performance, especially in terms of MSE, whereas CpG-level data required less features (Supplementary Table 1).

### The Effect of Limiting the Number of Features

**FIGURE 2:**
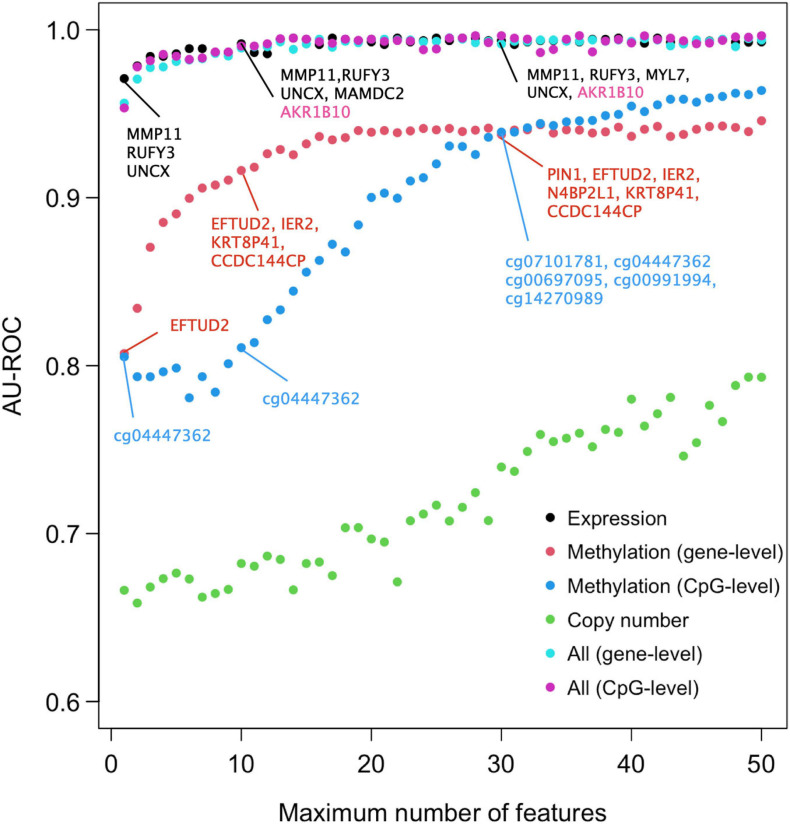
Predictive accuracy of the omics profiles and their combinations when the maximum number of features was limited. The points are average AU-ROC values over the 10 CV rounds in nested CV. Example feature sets from omics data are shown at limits *x* = 2, 10, and 30. The gene lists contain the features that were selected by Lasso in all the 10-fold at that limit. Expression data and integrated data share most genes in common. Black color indicates genes that were selected by both gene expression and integrated data, the pink color those genes that were selected by integrated data only. Note that the top genes of the two types of integrated data are the same. No copy number features were selected in all the 10 CV folds (no robust CNV features). See Supplementary Figure 4 for the version of ROC and PRCs with SEMs included.

We next studied the effect of limiting the number of features of the Lasso model on its predictive accuracy, with the aim to investigate what are the minimal panels of biomarkers that could cost-effectively distinguish DCIS cases from IBC. A feature number limit from 2 to 50 was imposed on each data type separately and in combination, and for each limit, 10-fold nested CV was applied to investigate the classification accuracy of the Lasso models with limited number of features. Notably, already two gene features provided an almost perfect AU-ROC of 0.95 when using the expression data only (Figure 2), indicating that sparse models enable accurate classification. However, the variability of the AU-ROC decreased when using the feature limit higher than 12 (Supplementary Figure 4), suggesting that the additional gene features make the classifier more stable.

When considering AU-ROC, the CpG methylation model performed initially worse, when compared to the gene-level methylation model, but after 30 CpGs its classification accuracy increased (Figure 2). The variability of the classification accuracy was also lower with the CpG-level model compared to the gene-level methylation model. These results suggest that when the variance of individual CpGs is large, the model cannot make reliable classification using only a small number of CpG features. Since the gene-level methylation signature consists of many CpGs collapsed to single genes, its variance tends to be smaller due to measurement noise being canceled out in the collapsing process. When considering AU-PRC, the gene-level methylation model remained slightly better than the CpG model across all the feature numbers (Supplementary Figure 4), and it also led to increased sensitivity of the multi-omics model, comparable to that of the gene expression only model (Supplementary Figure 5).

Since the features were selected in 10-fold nested CV at each feature number limit, the model may identify in total more features than the limit, since the different CV folds may select different features. Figure 2 lists as examples features that were selected in all the 10 CV folds, suggesting they are robust to training data subsampling and therefore likely to present robust classifying features. Such robust features could not be identified from the copy number data. DNA methylation profiles identified genes that are distinct from those identified using the gene expression data, both when using the gene-level or CpG-level methylation data (and the corresponding genes). However, a total of four genes (MMP11, RUFY3, UNCX, and MAMDC2) were selected using both versions of the integrated data; these are exactly the same genes Lasso model identified when using the gene expression data only and the feature limit of 2, further suggesting that transcriptomics alone leads to sparse and accurate signatures.

### Identification of Repeatedly Misclassified DCIS Cases

We next investigated whether the multi-omics data and the classification models could identify those DCIS-labeled samples with a potentially higher likelihood for progressing to an invasive state. Even if these DCIS samples have been originally labeled as DCIS in the diagnostic classification, they may still possess molecular changes that promote invasion later in time. In this analysis, we used Lasso and RF models, together with gene expression and CpG methylation profiles, due to their overall good performance. We considered for further investigation those DCIS cases in the training cohort that were repeatedly misclassified by these model–data combinations more than once (Table 1). Misclassification by one model–data combination may represent merely technical noise.

**TABLE 1:** Misclassified training samples when using various classification models and omics data.

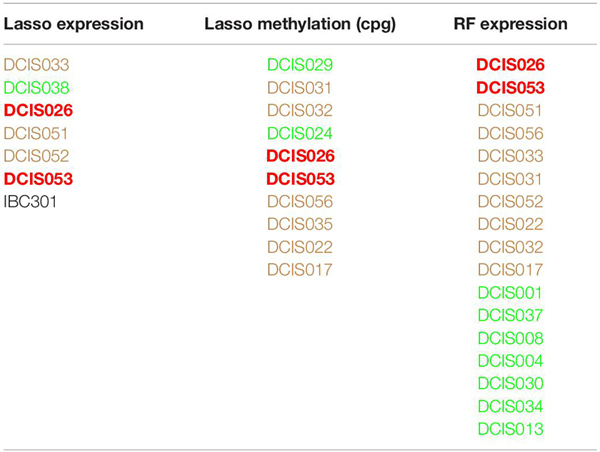

*Color coding indicates the number of times any of the 40 DCIS cases were misclassified as IBC in the training data; green, once; brown, twice; red, three times. CpG methylation data with the RF model was not used in these analyses since it misclassified a total of 34 DCIS cases, which was considered too many. We considered for further investigation only those DCIS cases in the training cohort that were repeatedly misclassified by these model–data combinations more than once.*

Out of the 40 DCIS cases, there were 19 lesions that were always correctly classified, and 11 DCIS cases were misclassified once, whereas eight and 2 DCIS cases were misclassified two or three times, respectively. We next applied so-called pseudo-labeling, where the repeatedly misclassified DCIS cases were relabeled as IBC, and then trained a new Lasso model with nested CV. Notably, such pseudo-labeling slightly increased the AU-PRC levels in the training cohort, while the AU-ROC levels remained similar to those with the original class labels (Supplementary Table 4). The multi-omics patterns provide evidence that these DCIS cases have molecular signatures more similar to the IBC cases and may have an increased likelihood to progress to an invasive disease stage.

### The Most Robust Genes for Classification

**FIGURE 3:**
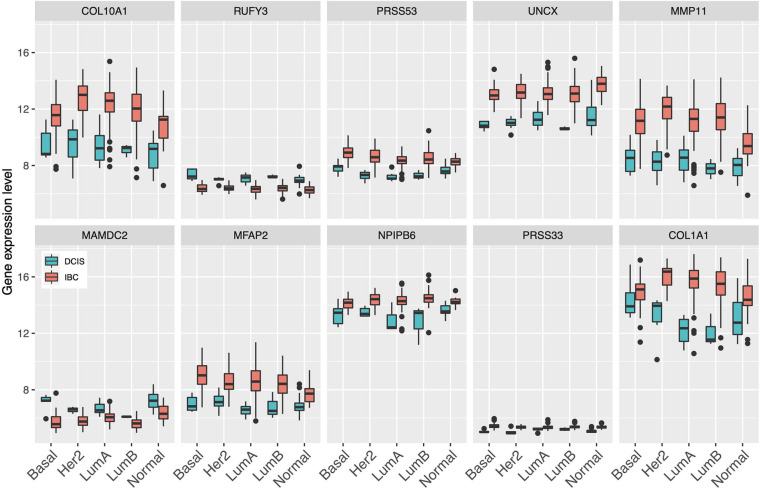
Expression levels of the select 10 genes across the established breast cancer subtypes. Basal, basal-like; HER2, HER2-enriched; LumA, luminal A; LumB, luminal B; Normal, normal-like.

Since gene expression was found to be generally the most predictive among the single omics data, we next identified the set of common genes selected by both the Lasso and SVM models using the gene expression features alone. We further required that a gene needs to be selected in more than 50% of the CV folds (i.e., more than five out of 10-folds), with the aim to guarantee robust and stable feature selection. In total, we found 10 such common and robust genes identified as robust risk signature. Notably, each of the 10 genes had a similar direction of differential expression between the DCIS and IBC classes across the established breast cancer subtypes (Figure 3), suggesting that they provide subtype-agnostic markers for breast cancer risk prediction.

**FIGURE 4:**
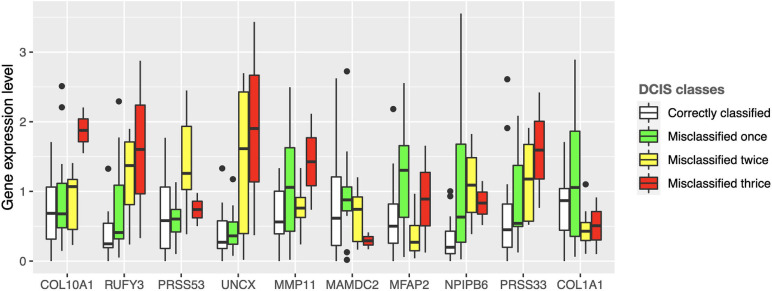
Expression values of the 10 genes across the DCIS cases repeatedly misclassified as IBC. The bars show absolute deviance from the median expression level of the correctly classified DCIS class for each gene. The median level of the correctly classified DCIS class was subtracted from the other classes to better show differential expression levels. Supplementary Figure 10 shows the expression differences before subtracting the median level of the correctly classified DCIS class.

Interestingly, there were marked differences in the expression levels of the 10 genes across the DCIS cases misclassified as IBC (Figure 4). For instance, RUFY3, UNCX, PRSS33, and COL10A1 showed an increasing trend of absolute expression changes between the DCIS cases as a function of the number of times the DCIS samples were misclassified by the models. This further demonstrates the molecular information captured in the expression profiles. Furthermore, based on the expression levels of the 10-gene signature, most of the sure DCIS cases that were always correctly classified were clustered together, whereas the repeatedly misclassified DCIS cases were scattered around in the unsupervised hierarchical clustering dendrogram (Supplementary Figure 6).

**FIGURE 5:**
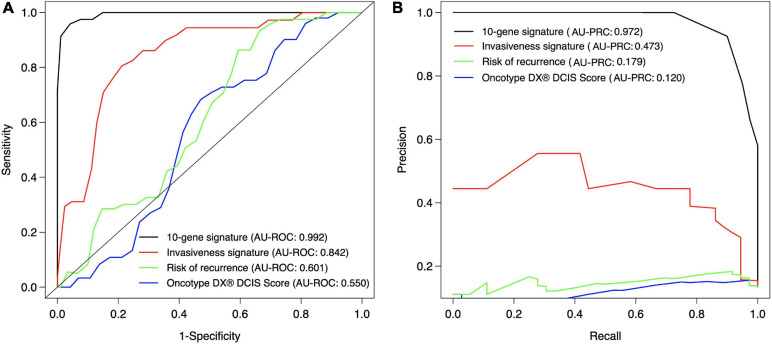
Classification accuracy of the 10-gene signature against existing scores. **(A)** ROC, **(B)** PRC. Each signature was calculated based on the training patient cohort. The ROR score is based on the PAM50 genes (Parker et al., 2009), invasiveness score is based on 64 invasiveness related genes (Anastassiou et al., 2011), and DX^®^ DCIS score based on seven genes (Solin et al., 2013). The expression values of the 64 genes were converted to z-score over each gene, and the average z-score was used as the invasiveness score for each sample. The original DX^®^ DCIS score was based on qPCR data, but here it was applied to microarray gene expression data. The curves show the mean sensitivity and specificity over 10 CV folds in the training cohort. See Supplementary Table 5 for the SD of the AU-ROC and AU-PRC values.

We next compared the classification accuracy of the 10-gene Lasso model against three existing risk signatures relevant for breast cancer progression: ROR (risk of recurrence), the invasiveness score (64-gene signature) and seven-gene DX^®^ DCIS score (see Methods). Our results showed that none of these risk scores was able to accurately distinguish between DCIS and IBC cases in our training cohort (Figure 5). In particular, using the default Lasso cutoff of 0.5, both the ROR and invasiveness score always classified all the DCIS lesions as IBC, whereas the DX^®^ DCIS Score classified all the IBC cases as DCIS (Supplementary Table 5). There were three common genes between the 64-gene invasiveness signature and our 10-gene signature (COL1A1, COL10A1, and MMP11), hence explaining its higher classification accuracy compared to ROR.

### Validation Set Results

**FIGURE 6:**
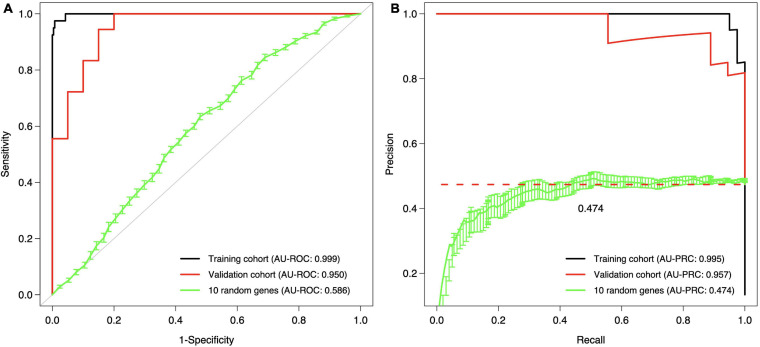
Validation and training cohort accuracies of the 10-gene signature. **(A)** ROC, **(B)** PRC. The Lasso model was first estimated based on the full training dataset using the 10 genes as features, and then the estimated model was applied to the validation cohort. The training cohort model accuracy is overoptimistic as no cross validation was used and the training and test data are the same; see Figure 5 for cross-validated training cohort model accuracy. For comparison, we randomly selected 10 genes 100 times, estimated 100 Lasso models in the training cohort, and then tested these random gene classifiers on the validation cohort. The 10 random gene curve shows the average performance of the random classifiers, and the error bars show the standard error of the mean (SEM). In panel **(B)**, the dashed horizontal line corresponds to a theoretical random classifier with AU-PRC = 0.473.

The final step was to validate the 10-gene signature on an external data set, the Curie Cohort, with the aim to investigate whether the DCIS classification model generalizes also beyond the training cohort to an independent validation dataset. The Lasso model of 10 genes estimated in the full training dataset was shown to provide highly accurate classification between the DCIS and IBC cases also in the validation dataset (Figure 6). Notably, both the AU-ROC and AU-PRC values dropped only slightly from the training to the validation cohort, further demonstrating the reliability and robustness of the classification model based on the 10-gene signature. However, we note that the default classification cutoff of 0.5 was not optimal in the validation data, but instead smaller thresholds led to better classification accuracy (Supplementary Figure 7). This is likely due to the differences between the microarray gene expression data (training cohort) and RNA-sequencing data (validation cohort). Although we performed z-scoring to unify the scales, it cannot correct for all the distributional differences between microarray and RNA-sequencing data.

**FIGURE 7:**
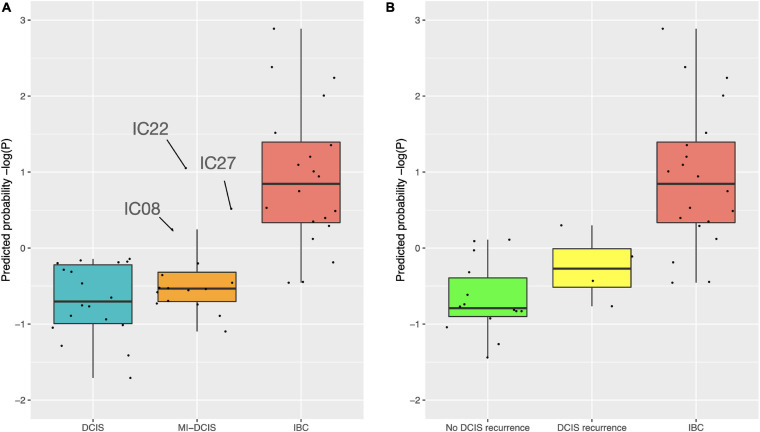
Predicted class probabilities of the validation cohort samples. **(A)** The probabilities were calculated based on the 10-gene Lasso model estimated on the full training data. The validation cohort included 18 pure DCIS cases, 20 IBC cases, and 16 micro-invasive (MI) DCIS cases. Note: y-axis is log-scaled for better visualization. The non-logged version is shown in Supplementary Figure 8. **(B)** The probabilities were calculated based on the six microenvironment related genes. The validation cohort included 14 DCIS cases without recurrence, four DCIS recurrence cases, and 20 IBC cases. The y-axes are z-scaled for better comparability of the prediction probabilities. The horizontal lines in the boxplots indicate median values, the whiskers the interquartile range (IQR) of the cases in each class, and the error bars show cases withing 1.5xIQR, while the remaining cases are considered outliers.

We further tested how the model predicts the microinvasive (MI) DCIS cases in the validation cohort to explore whether the 10-gene signature could also distinguish the MI cases from pure DCIS and IBC cases. Interestingly, the classification probabilities of the MI DCIS cases were in between the pure DCIS and IBC classes but remained significantly closer to the pure DCIS cases (Figure 7, left). However, there was a relatively large variability in the distribution of the predicted probabilities also within the classes, showing individual variability in the risk scoring based on the 10-gene signature. This suggests that there are molecular-level changes in these genes between the classes of pure DCIS, DCIS-MI, and IBC lesions. Interestingly, there appeared to be three outlier cases in the DCIS-MI class with the classification probability comparable to that of the IBC cases. The six genes that were related to the microenvironment (COL10A1, COL1A1, MFAP2, PRSS33, PRSS53 and MMP11) showed higher prediction probability in the recurrent DCIS cases, compared to DCIS without recurrence, and these became close to those of the IBC cases (Figure 7, right).

**FIGURE 8:**
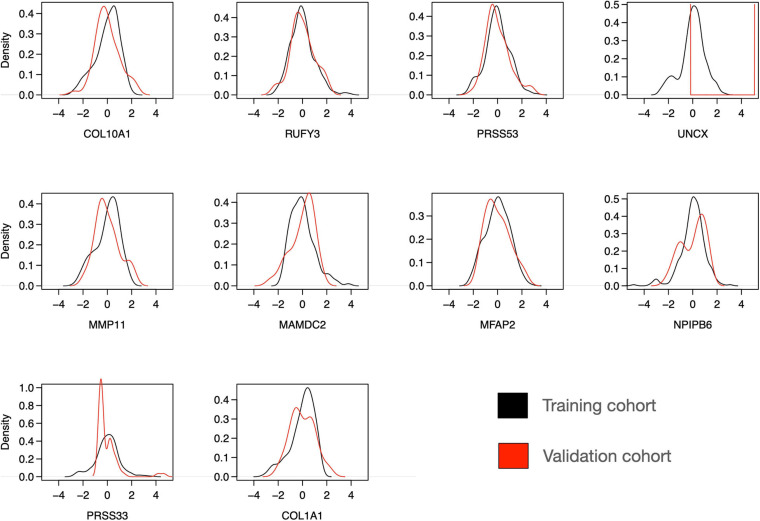
Distribution of expression levels of 10 selected genes over all the individuals in the training and validation cohorts. The scales between the microarray (training cohort) and RNA-seq (validation cohort) data were harmonized using z-score normalization over each gene separately in the training and validation datasets. Note: the y-axis density ranges differ between the panels.

To further investigate the features of the sparse Lasso model, we plotted the expression distributions of the 10 genes on both the training and validation cohorts (Figure 8). After z-scoring, most of the genes showed similar distributions, except for UNCX and PRSS33. In particular, for UNCX, there were only two distinct expression values in the test RNA-seq data, and 53 out of 55 cases (96%) corresponded to zero expression in the original expression data before z-scoring. There were also marked differences in the expression levels of the 10 genes across the three disease classes of the validation cohort (Supplementary Figure 9), mostly differentiating IBC cases from DCIS and DCIS-MI, even though the differences were not as clear as in the training cohort (Figure 3). However, regardless of these technical and biological differences between the training and validation cohorts, the 10-gene signature provided accurate classification performance in both of the datasets, further demonstrating its robust behavior. Taken together, these results indicate that the 10-gene signature can reliably identify those DCIS cases that are less likely to progress to invasive disease.

## Discussion

In our multi-omics classification analysis between DCIS and IBC, we found that the gene expression-based model features led to the highest classification accuracy alone; however, methylation data provided a complementary source of predictive signal, and it improved especially the sensitivity of correctly classifying DCIS cases, which is important for clinical application of risk signatures. No better prediction results could be obtained with any of the two-data combinations, and the gene expression data was always required for the best prediction results, indicating its high predictive contribution. Due to the challenges of acquiring fresh frozen DCIS tissue, the number of DCIS cases was much smaller in the training cohort, compared to the IBC cases. We used several computational approaches to take into account such unbalanced classification setting: (i) we used several evaluation metrics to provide multiple views into the predictive performance of the models, including precision–recall analysis, which is often considered more suitable for the unbalanced classification problem; (ii) we included only those omics features in the signature that were robustly identified using multiple algorithms and across several CV rounds; (iii) we carried out the pseudo-labeling approach to investigate whether relabeling of some of the recurrently mis-classified cases could increase the predictive performance of the model and reveal potentially high-risk DCIS cases; and finally (iv) we validated the predictive power of the signature in an external validation cohort with more balanced classes.

Previous studies have found only moderate genomic and epigenomic differences between DCIS and IBC (Ma et al., 2003; Hannemann et al., 2006; Fleischer et al., 2014; Abba et al., 2015; Pang et al., 2017). In this study, we identified 10 genes using both the Lasso and SVM models that were selected in >50% of the CV rounds, indicating their robust behavior for classification between DCIS and IBC cases. We also found that these genes were differentially expressed between DCIS and IBC across all the breast cancer subtypes (Figure 3). One should interpret such gene lists with caution, however, as there may be other gene combinations with similar predictive power due to the correlated nature of the gene expression profiles among genes in the same pathways or biological processes. Nevertheless, the genes were selected by two independent methods, which increases the robustness of their biological signal. The 10-gene signature was also validated in independent test data (Curie cohort), where the transcriptional profiling was done with RNA-seq. The high classification accuracy observed for the 10 genes, originally identified using gene expression microarrays, further demonstrates the robustness of the signature, although there remained some variability that is beyond z-score normalization (Figure 8). We also note that the 10-gene signature was not able to predict recurrence in the validation cohort, as expected, since the genes were selected specifically for distinguishing between DCIS and IBC classes, not the progression of DCIS cases.

The comparison between our 10-gene signature and traditional breast cancer risk scores further demonstrated the added value of our 10-gene markers especially for the accurate DCIS classification (high sensitivity). We note that ROR is mostly affected by proliferation, and it is highly associated with breast cancer subtypes (Parker et al., 2009). Our results therefore indicate that proliferation may not be very important when distinguishing between DCIS and IBC cases. However, the invasiveness score has previously been found highly associated with cancer cell motility and invasiveness of several cancer types, including non-epithelial cancers such as neuroblastoma (Anastassiou et al., 2011). This should make it a competitive biological marker to classify DCIS and IBC. Our results showed that the invasiveness score achieved a relatively high classification accuracy (AU-ROC 0.842), but not as high as our 10-gene signature (AU-ROC 0.992). The 64 genes included in the invasiveness signature had three genes in common with our 10-gene signature (COL1A1, COL10A1, and MMP11). Since we demonstrated that already two genes can give a relatively high AUC (Figure 2), and MMP11 is one of the selected genes when the feature limit was two, the higher performance of the invasiveness signature was as expected. However, the extended set of 10 genes provided increased performance especially for classification sensitivity. Furthermore, measuring 10 genes is more practical than measuring 64 genes using, for instance, qPCR-based clinical assays.

Many of the genes included in the model have previously been identified as differentially expressed between DCIS and IBC (Lesurf et al., 2016), but there are also some novel genes. Out of the 10 genes, six are related to the tumor microenvironment (COL10A1, COL1A1, MFAP2, PRSS33, PRSS53, and MMP11), and these genes showed predictive power for recurrent DCIS (Figure 7), although its added value for clinical practice remains to be investigated on a larger series. COL10A1, COL1A1, and MFAP2 are constituents of the extracellular matrix remodeling, which is an important process in breast tumor invasion and tumor cell dissemination (McSherry et al., 2007). Overexpression of the genes encoding these proteins is associated with poor breast cancer survival, and MFAP2 has been shown to promote cell proliferation, migration, invasion, and epithelial to mesenchymal transition (Wang et al., 2018; Liu et al., 2018; Zhang et al., 2020). MMP11 is a proteinase that is involved in extracellular matrix degradation directly by degrading collagen IV and indirectly by inhibiting the alpha1-proteinase inhibitor (Pan et al., 2003; Motrescu et al., 2008. MMP11 has been characterized extensively for its role in breast cancer and has been shown to be a predictive factor for tumor invasiveness, hence serving as positive control here (Ahmad et al., 1998; Zhang et al., 2016). In contrast, the roles of the serine proteases PRSS33 and PRSS53 have been less investigated in cancer progression, but there are indications that PRSS33 may play a role in tumor cell invasion (Jeong et al., 2016).

The remaining four genes in our gene list are not directly associated with the microenvironment. For instance, RUFY3 is involved in F-actin-enriched protrusions from the cell surface and it has been shown to be involved in gastric cancer cell migration and invasion (Wang et al., 2015). This gene, however, shows paradoxical expression in our training data with higher expression in DCIS than in invasive samples (Figure 3). In the validation cohort, however, the expression levels of RUFY3 were as expected in the DCIS and IBC classes (Supplementary Figure 9), especially when focusing the recurrent DCIS cases (Supplementary Figure 11). UNCX was another gene with distinct expression distribution between training and validation data. It is a homeobox transcription factor that has been associated with acute myeloid leukemia (Daniele et al., 2017). MAMDC2 is a known tumor suppressor involved in glycosaminoglycan binding (Lee et al., 2020), whereas NPIPB6 has not previously been associated with cancers to the best of our knowledge. We note that the 64 genes included in the invasiveness signature are mainly related to epithelial–mesenchymal transition (EMT) (Anastassiou et al., 2011). The improved performance of the 10-gene signature indicates that the molecular changes from DCIS to IBC not only are related to the EMT process but also involve other biological processes captured by the 10-gene signature. To further study the biological processes, larger DCIS cohorts will need to be collected beyond those in the current training cohort (Sweden, Italy, and Norway).

Since our analyses were performed across the molecular intrinsic subtypes of breast cancer, the identified genes can detect DCIS cases, regardless of their subtype. The genes therefore represent general invasion processes, while the subtype-specific tumor progression processes may be obscured. A major proportion of breast cancer samples are Luminal A, and this is also the case in the training cohort. We have previously shown that Luminal A DCIS and IBC are highly similar at a molecular level, while basal-like DCIS differ substantially from basal-like IBC (Bergholtz et al., 2020). Stratification by subtype prior to creating the models could yield different results and identify genes and biological processes relevant within each subtype, but this approach would, in our high-dimensional analysis, be limited due to rather low sample size of the current cohorts. We believe that a subtype-agnostic model should become more practical for a clinical application of the signature, avoiding the need for subtype classification of each DCIS case. Additional genes would need to be included, such as those in the PAM50 signature, if one wants to construct risk signatures separately for the established subtypes. Furthermore, many studies have found stromal difference between DCIS and IBC (Dabiri et al., 2013; Toss et al., 2020), and it would be interesting to investigate how these 10 genes are expressed in stromal component vs. other components using spatial gene expression profiling.

Our results of the classification analyses using the two options to represent DNA methylation (preselected enhancer and promoter CpGs related to breast cancer biology or PCA-derived gene-level methylation) suggests that few individual CpGs cannot capture enough variation for accurate prediction and that a certain number of CpGs (>30 features) are needed to represent a meaningful information identifying DCIS from IBC. Moreover, we observed that CpG-level methylation features show higher sensitivity than gene-level methylation features using the Lasso model. This result highlights the importance of both enhancer and promoter methylation for gene regulation in breast cancer. On the other hand, the gene-level methylation represents many CpGs for each gene and thus it captures more variation, but some important CpGs may be masked by the PCA summarization approach. Furthermore, classification made using only a few individual CpGs may be vulnerable to measurement noise, and this can be overcome by increasing the number of CpGs in the classifier. Using all the 450,000 CpGs led to a poor class prediction performance, likely due to model overfitting (data not shown). Since the optimal processing of DNA methylation data is still poorly understood, we hope these results will provide guidance for the community on how to use methylation features in predictive modeling, either alone or combined with other omics features.

We initially tested several classification algorithms, Lasso, SVM, and RF, which all supported the importance of multi-omics profiles for increased DCIS detection sensitivity (Supplementary Tables 1–3). The lasso-regularized model generally showed the best performance and was therefore selected to showcase the classification results, for instance, when limiting the maximum number of features in sparse predictive modeling (Figure 2). Compared to genome-wide measurements, such minimal predictive signatures may lead to more practical prediction models for clinical decision tools in the form of cost-effective signatures for economic implementation. As observed before, nested CV was found important to avoid selection bias and reporting of overoptimistic results about the predictive power of classifier (Ambroise and McLachlan, 2002; Varma and Simon, 2006). As a future research direction, we plan to make use of pathway information for mapping the predictive genes that may potentially lead to even more robust and accurate models using pathway-level biomarkers (Ben-Hamo et al., 2020; Madani Tonekaboni et al., 2020). While the present work focused solely on protein-coding genes, since this enabled better interpretation of the model results and easier integration among the three data types, recent work has shown the influence of non-coding gene expression on cancer progression (Bhan et al., 2017; Chi et al., 2019; Zhang et al., 2021). As a future development, it would be interesting to use also non-coding DNA or RNA as additional source of features in the classification between DCIS and IBC cases.

In conclusion, our results support the use of the 10-gene signature to reliably identify those DCIS cases that are less likely to progress to invasive disease and may therefore have potential for reducing the current overtreatment in breast cancer. Longitudinal follow-up data of the DCIS cases will be needed for prognostic validation of the signature in terms of its accuracy at identifying high-risk vs. low-risk DCIS cases, and to explore how many of the initially DCIS diagnosed cases will eventually progress to an invasive disease or become invasive recurrent.

## Data Availability Statement

The original contributions presented in the study are included in the article/Supplementary Material, further inquiries can be directed to the corresponding author/s.

## Ethics Statement

The studies involving human participants were reviewed and approved by approval numbers: 2016/433 (Oslo, Norway), PG/U-25/01/2012-00001497 (Milan, Italy), and 2005/118 (Uppsala, Sweden). The patients/participants provided their written informed consent to participate in this study.

## Author Contributions

HX analyzed the data, implemented the predictive models, prepared the figures, and drafted the manuscript. TL contributed to the data analysis and predictive modeling. HB existed risk scores. HB and TS interpreted the biological results. TF provided the methylation data process. LD and AV-S provided the validation data. LD, AV-S, and TA interpreted the results. TS and TA co-supervised the work. TA designed the study. All authors contributed to the article and approved the submitted version.

## Conflict of Interest

The authors declare that the research was conducted in the absence of any commercial or financial relationships that could be construed as a potential conflict of interest.
