# Supplementary material for: Multi-Omics Marker Analysis Enables Early Prediction of Breast Tumor Progression
Source: Front Genet. 2021 Jun 3;12:670749. doi: 10.3389/fgene.2021.670749 (PMC8209521; doi:10.3389/fgene.2021.670749)
Supplement: Supplementary file 1 [file Data_Sheet_1.pdf]

Supplementary Material

1 Supplementary Figures and Tables

1.1 Supplementary Figures

Supplementary Figure 1. The number of samples profiled with each omics technique. The common set of 299 samples with each three omics data available was used for the model construction in this study. NA, not available; DCIS, ductal carcinoma in situ; IBC, invasive breast cancer.

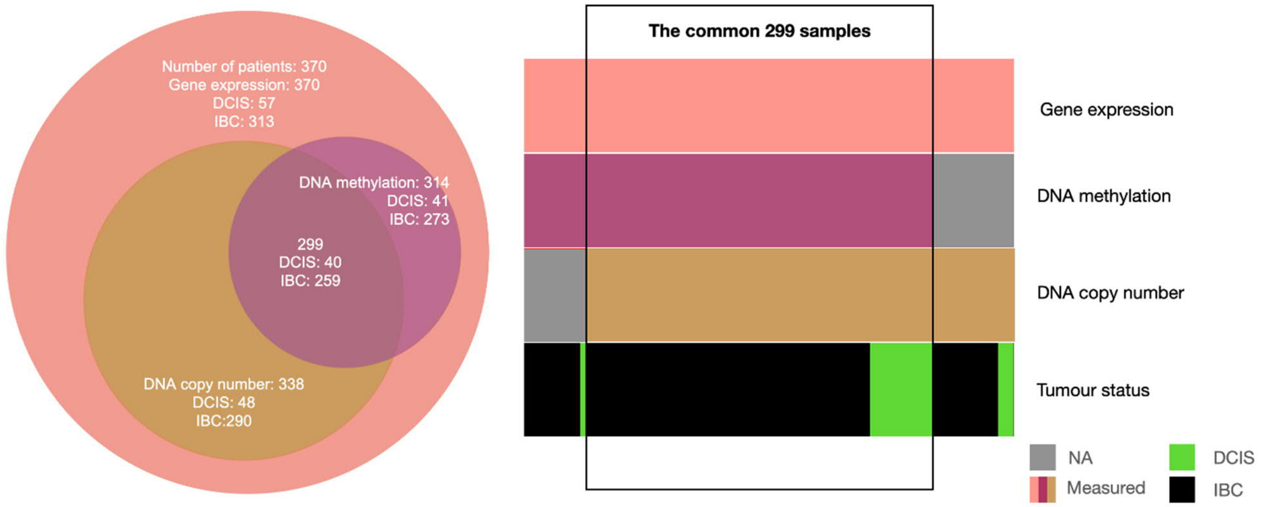

Supplementary Figure 2. Examples of evaluation metrics considered in the study. (A) ROC curve, where the red point indictes the default classification cutoff of 0.5 of the Lasso model. BA, balanced accuracy. The diagonal line corresponds to the random classifier with AU-ROC=0.5. (B) PRC of the same classification model. The dotted horizontal line corresponds to the random classifier with AU-PRC=0.0339.

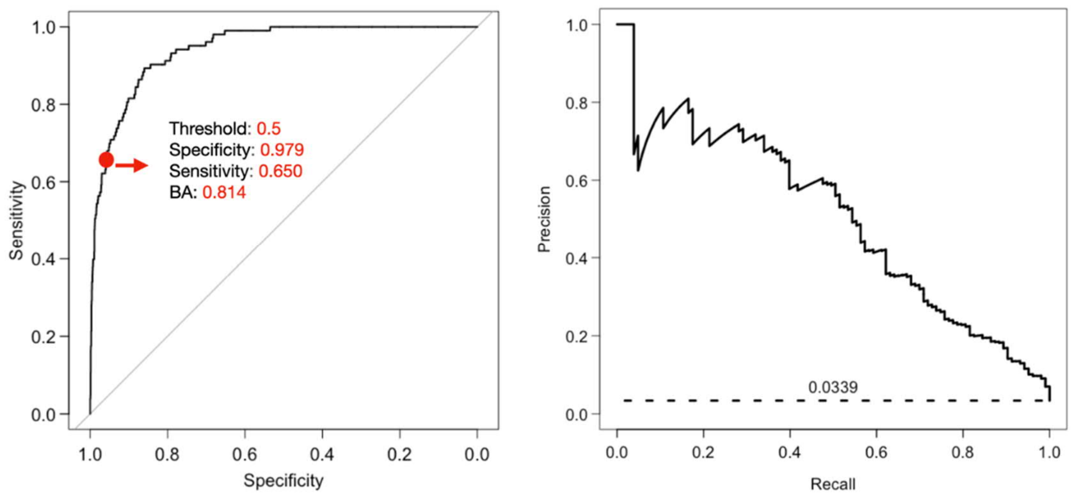

Supplementary Figure 3. ROC and PRC curves of the Lasso models when using multi-omics features, separately for each of the 10 CV rounds.

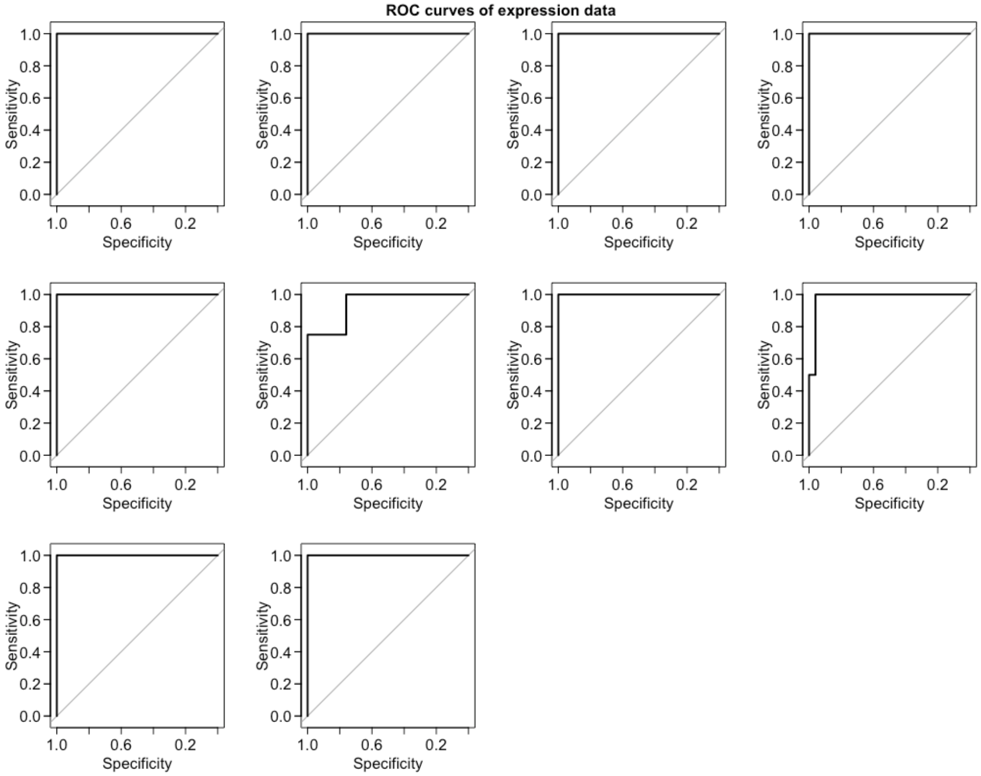

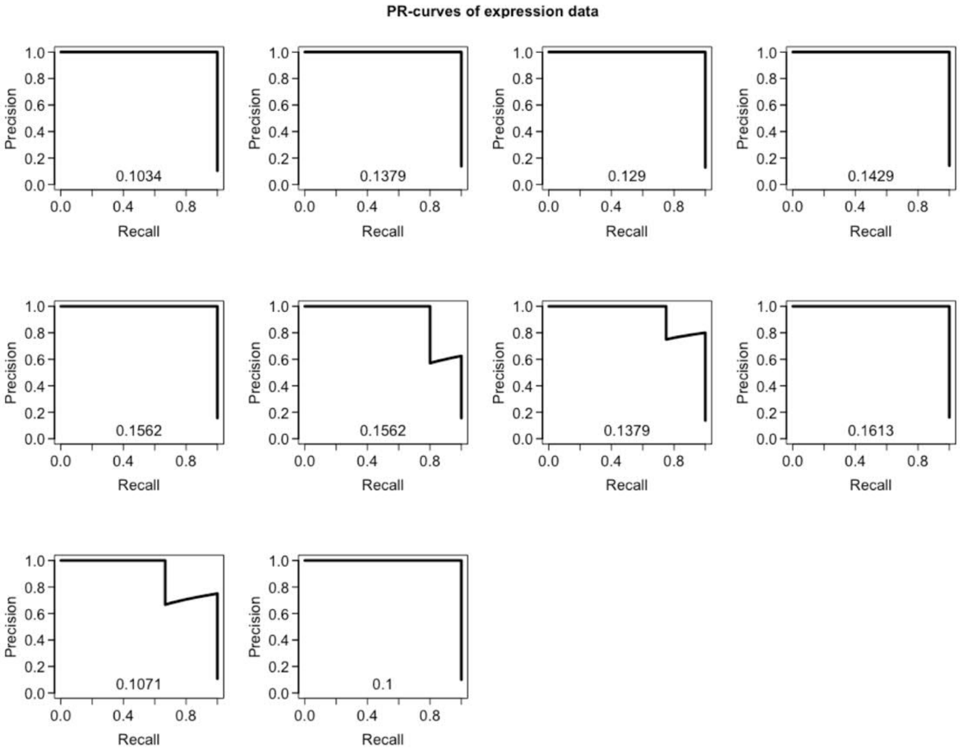

ROC curves of methylation data (gene-level)

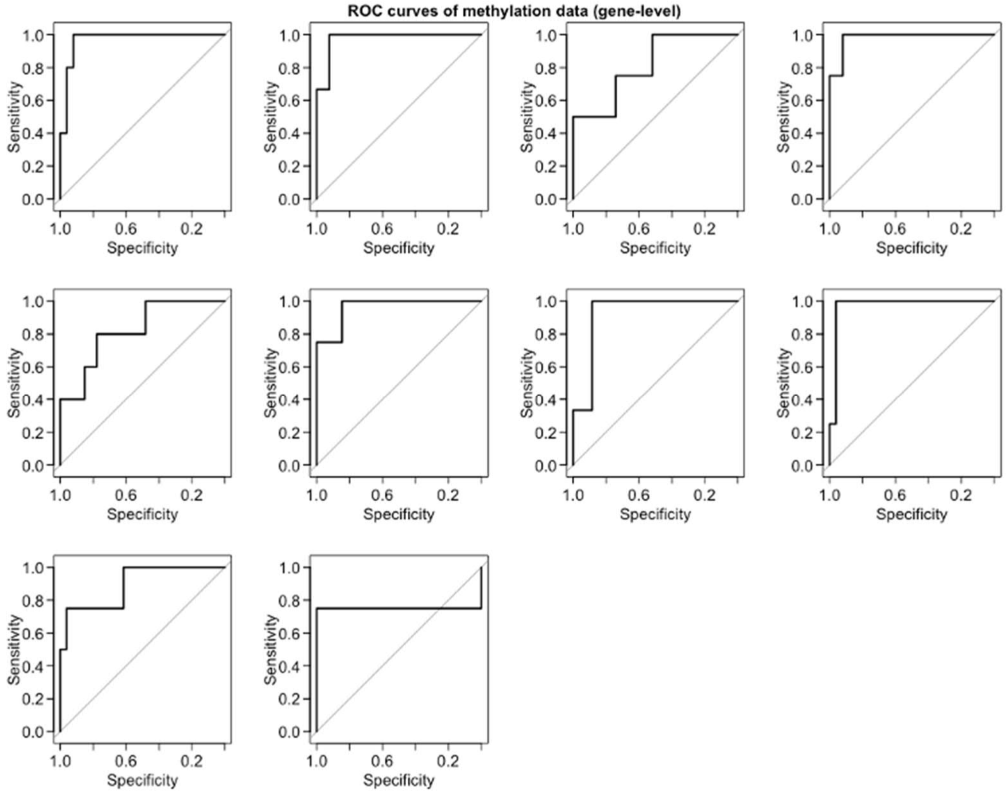

PR-curves of methylation data (gene-level)

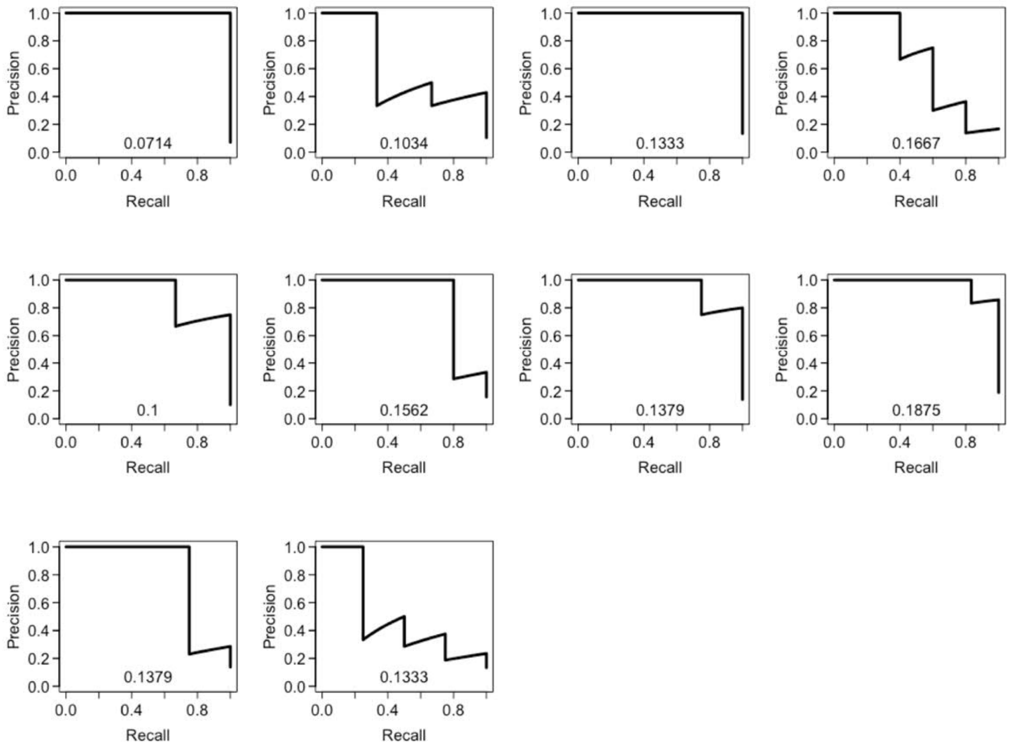

ROC curves of methylation data (CpG-level)

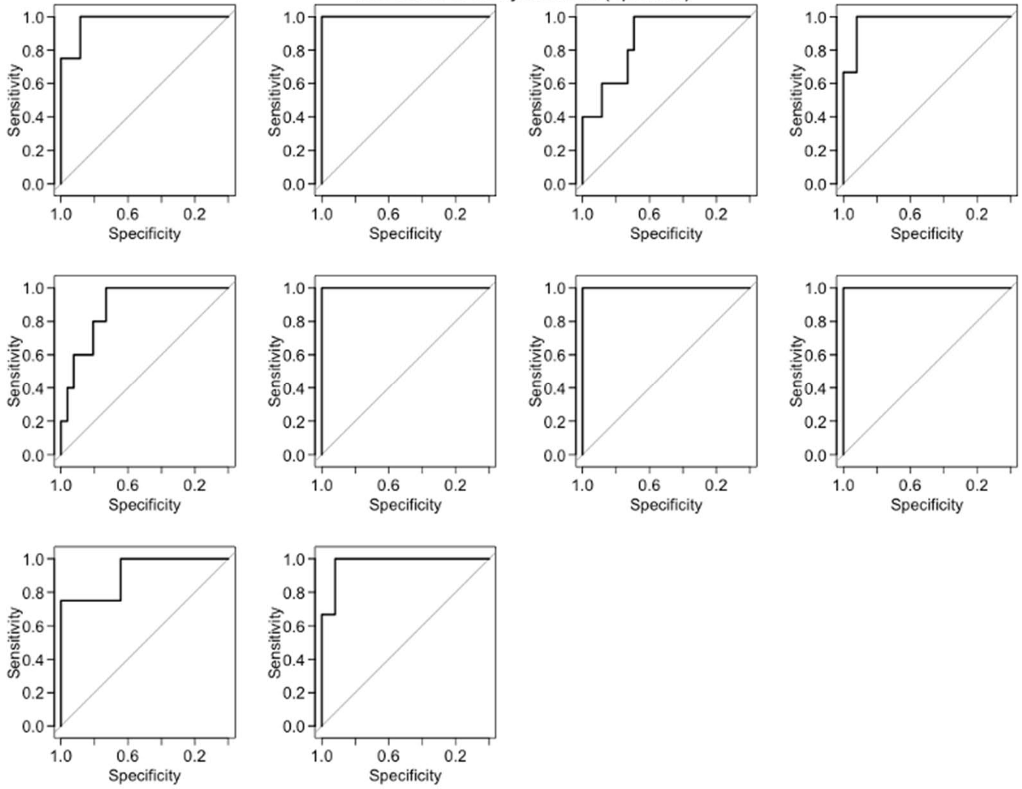

PR-curves of methylation data (CpG-level)

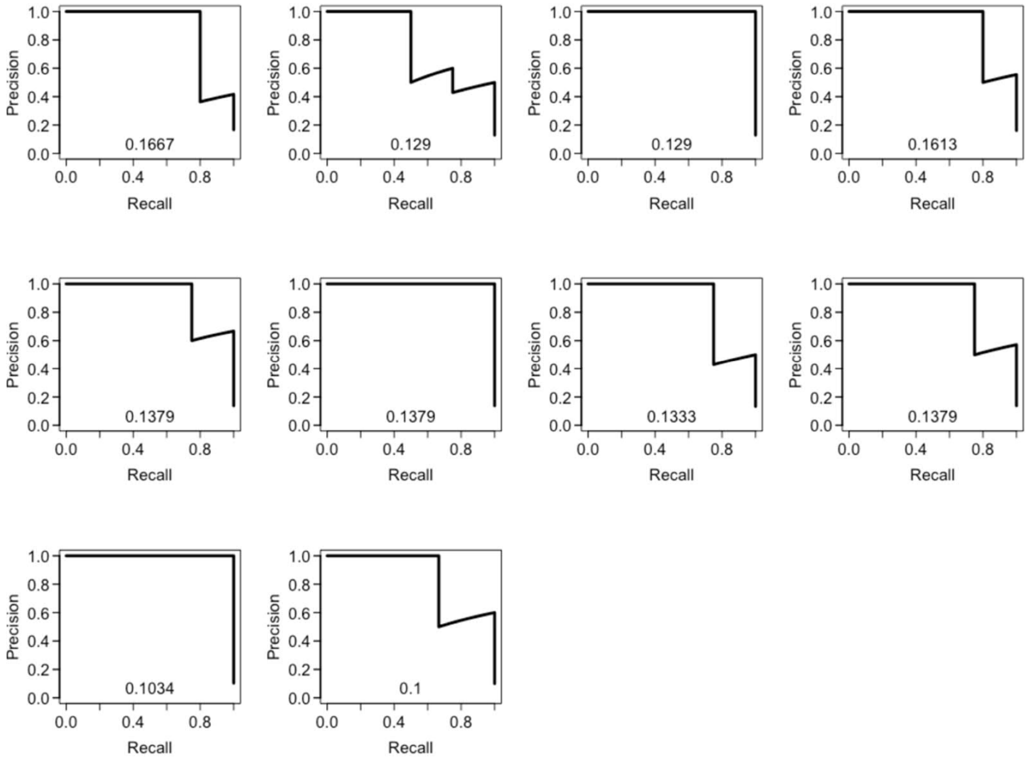

ROC curves of Copy number data

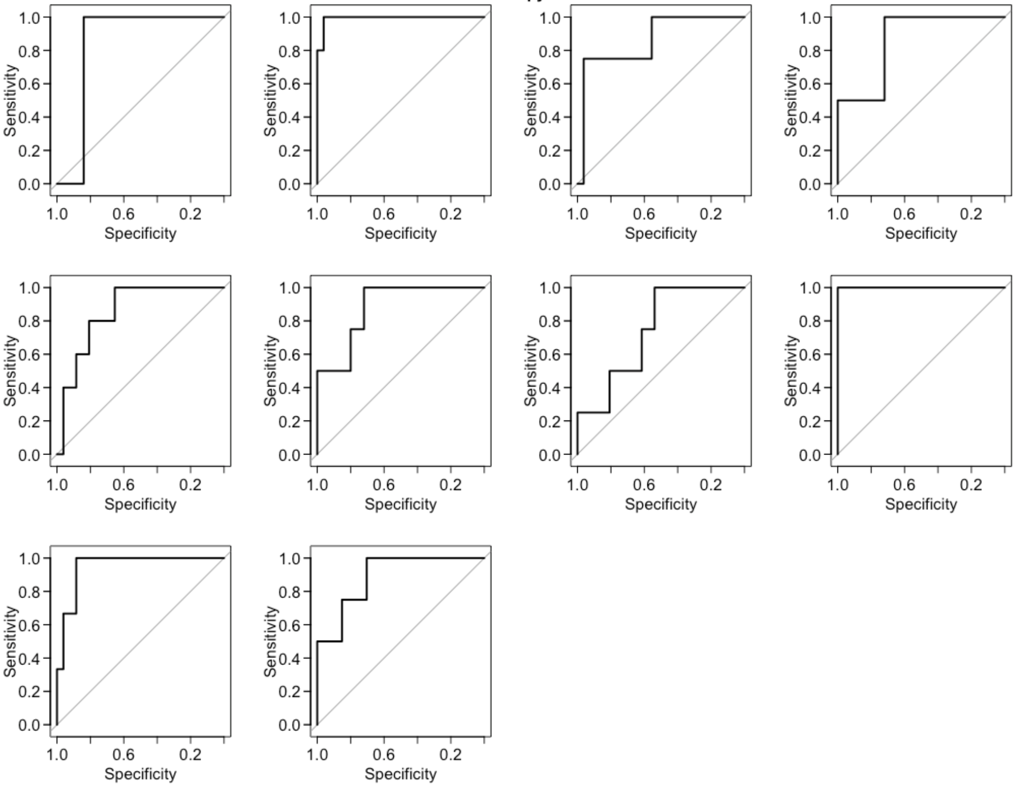

PR-curves of copy number data

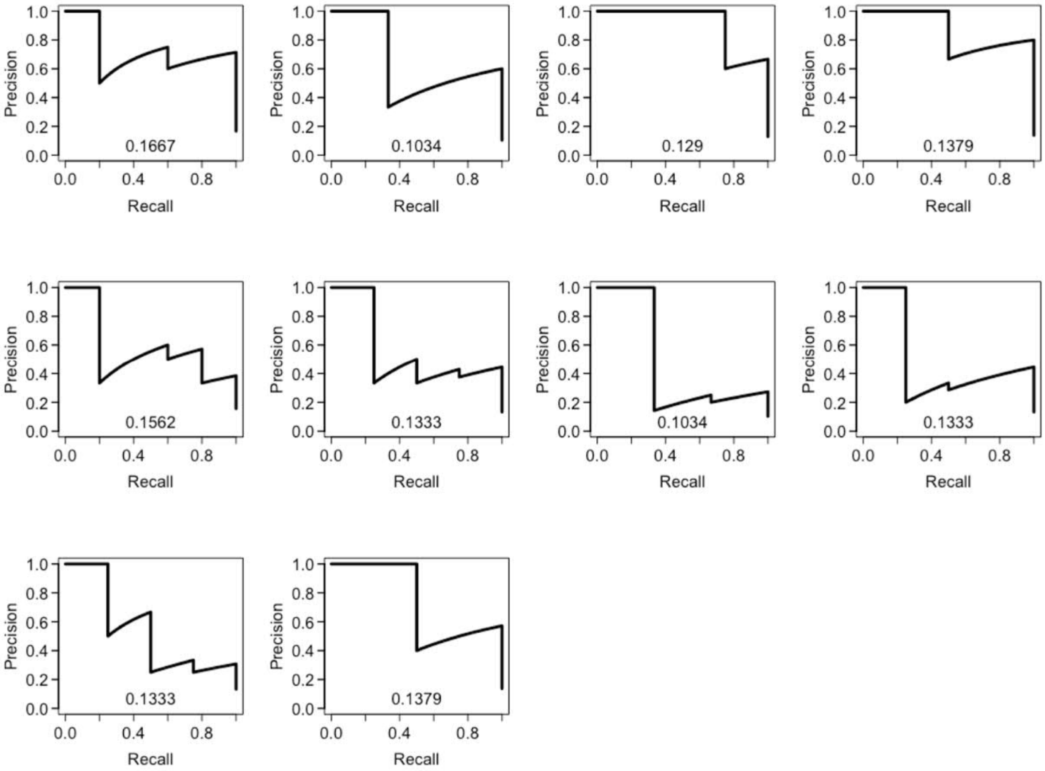

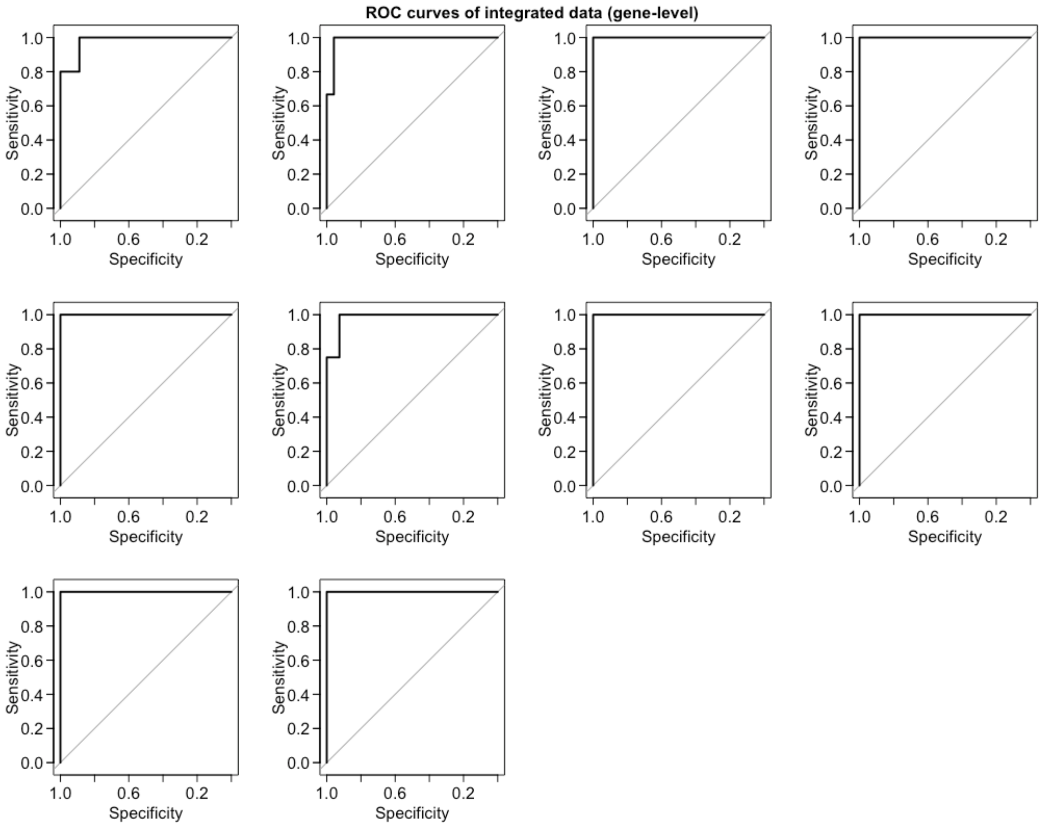
ROC curves of integrated data (gene-level)
Sensitivity
Specificity
0.0 0.2 0.4 0.6 0.8 1.0
1.0 0.6 0.2

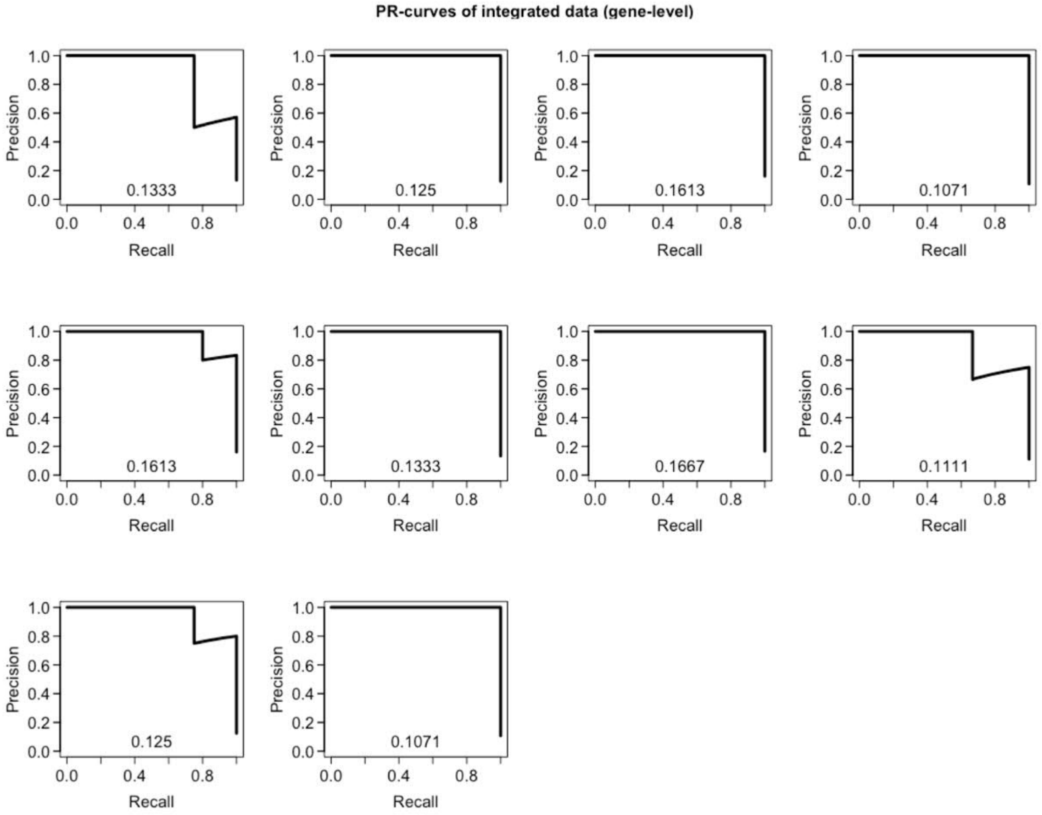
PR-curves of integrated data (gene-level)
Precision
Recall
0.0 0.2 0.4 0.6 0.8 1.0
0.0 0.4 0.8
0.1333
0.125
0.1613
0.1071
0.1613
0.1333
0.1667
0.1111
0.125
0.1071

ROC curves of integrated data (CpG-level)

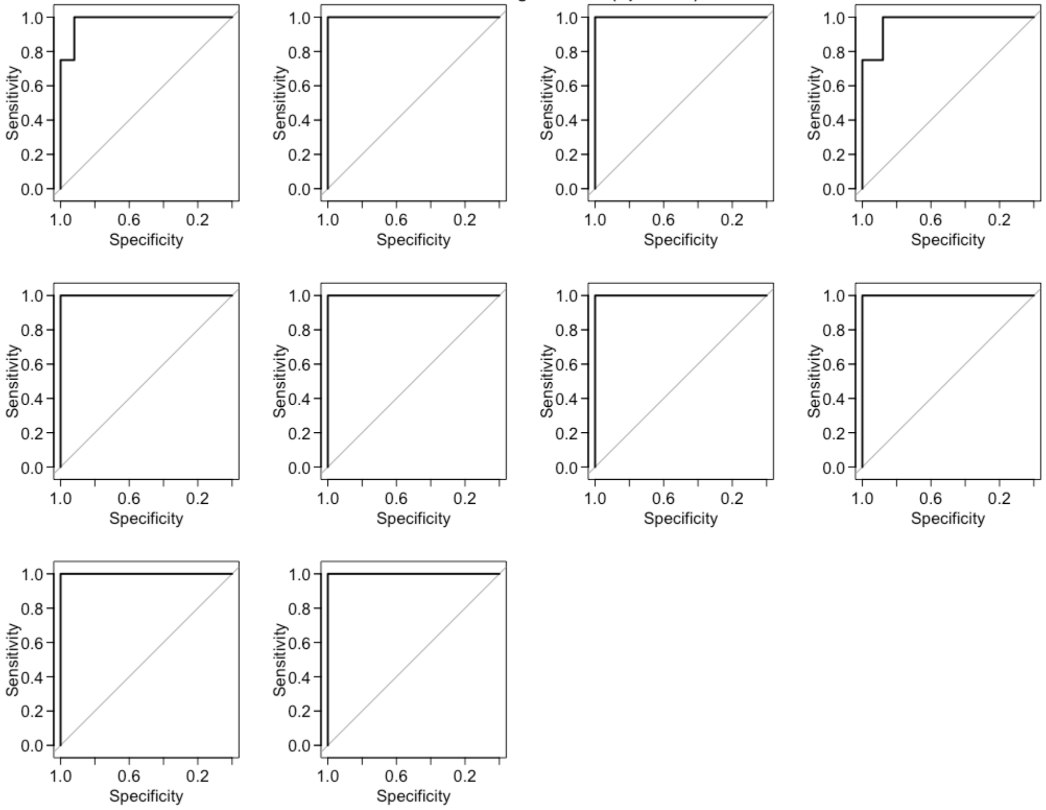

PR-curves of integrated data (CpG-level)

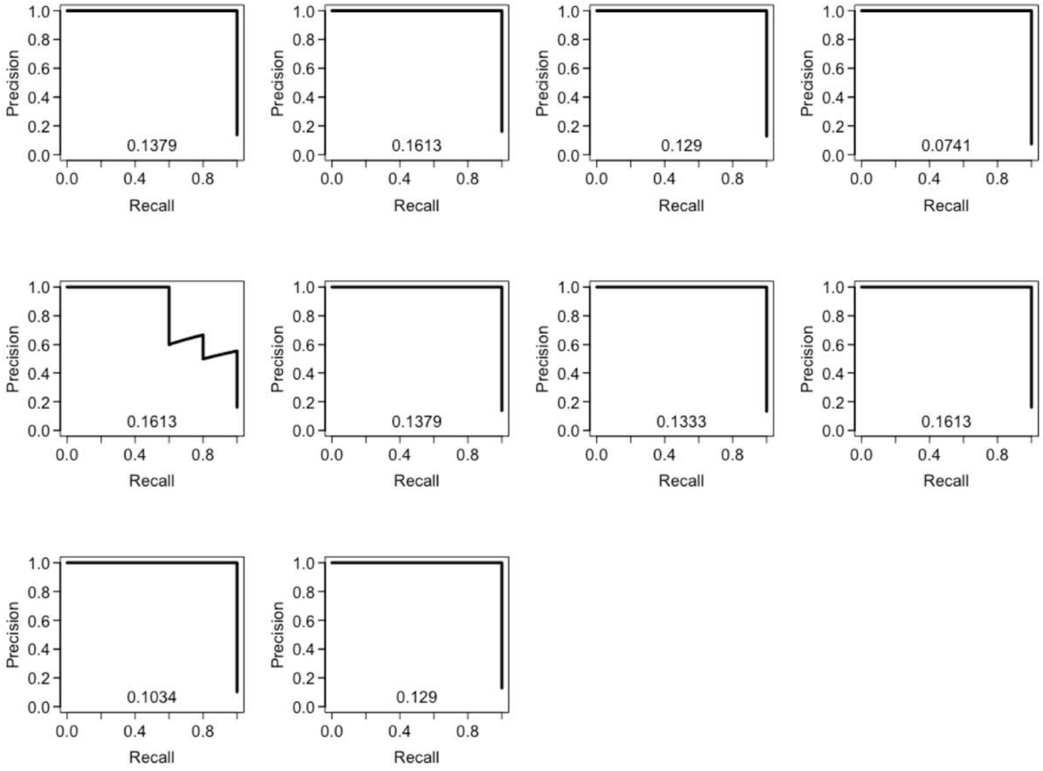

Supplementary Figure 4. Lasso model accuracy as a function of the maximum number of features. (A) ROC, (B) PRC. Error bars indicate the standard error the mean (SEM). See Figure 2 legend for further details.

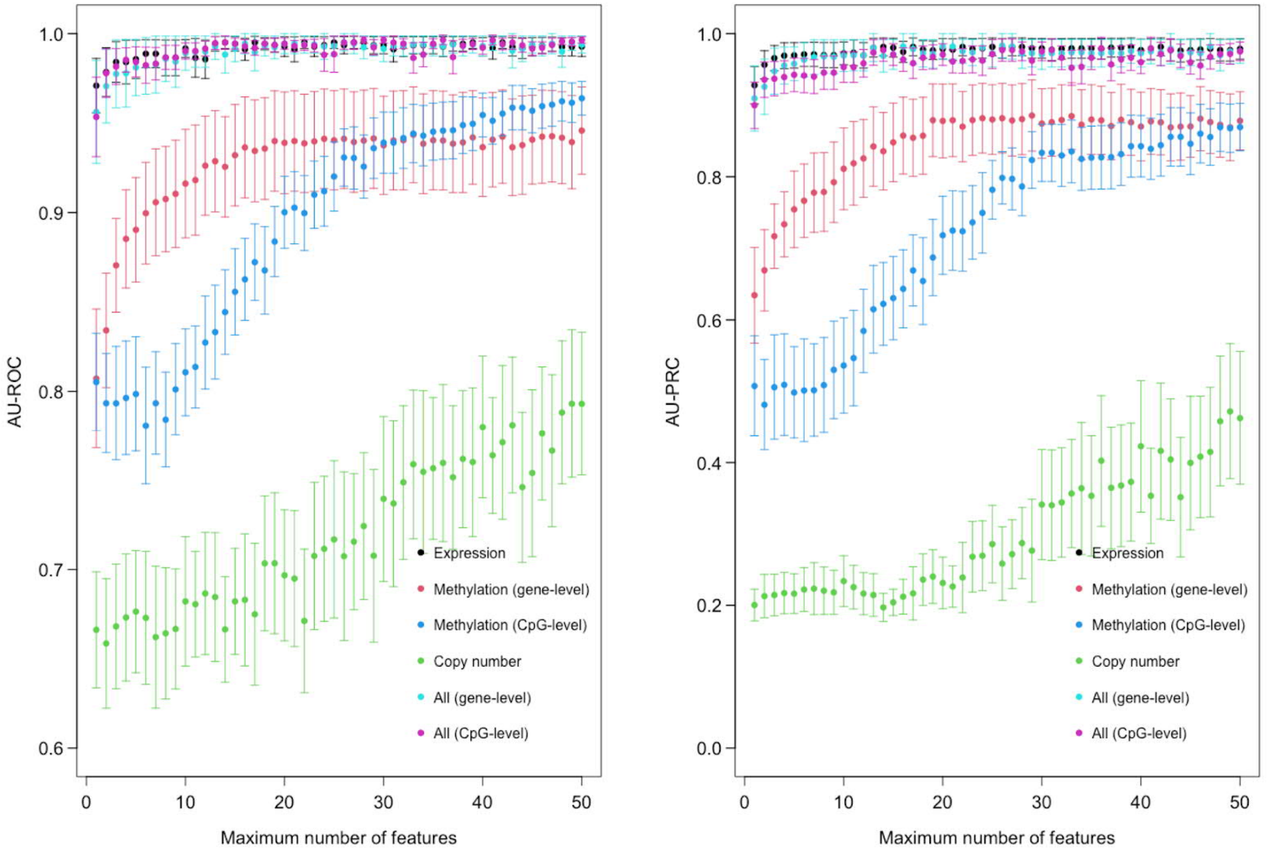

Supplementary Figure 5. (A) Sensitivity and (B) specificity of the Lasso model as a function of the maximum number of features. The specificity of most models is 1.0 (overlapping curves).

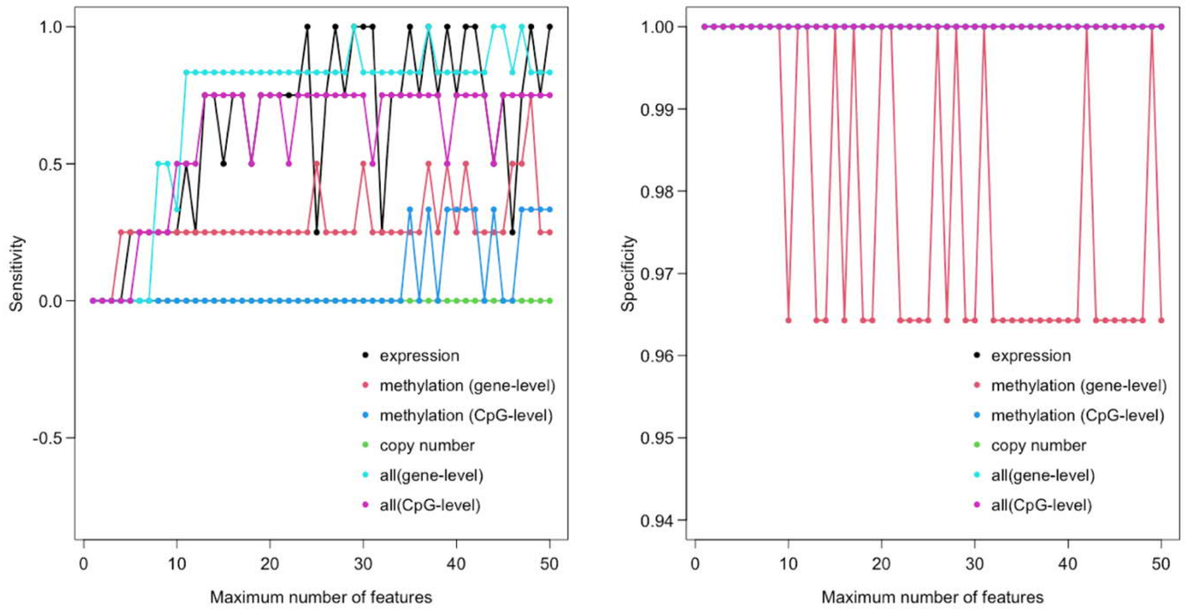

Supplementary Figure 6. Unsupervised clustering of the training cohort based on the 10-gene signature. (A) DCIS cases, (B) all the samples. Color-coding of the samples indicates breast cancer subtyping and how many times DCIS cases were mis-classified as IBC using various classification models and omics data. The clustering was performed with the complete hierarchical clustering algorithm using the ComplexHeatmap R-package, with Euclidean distance as the similarity metric.

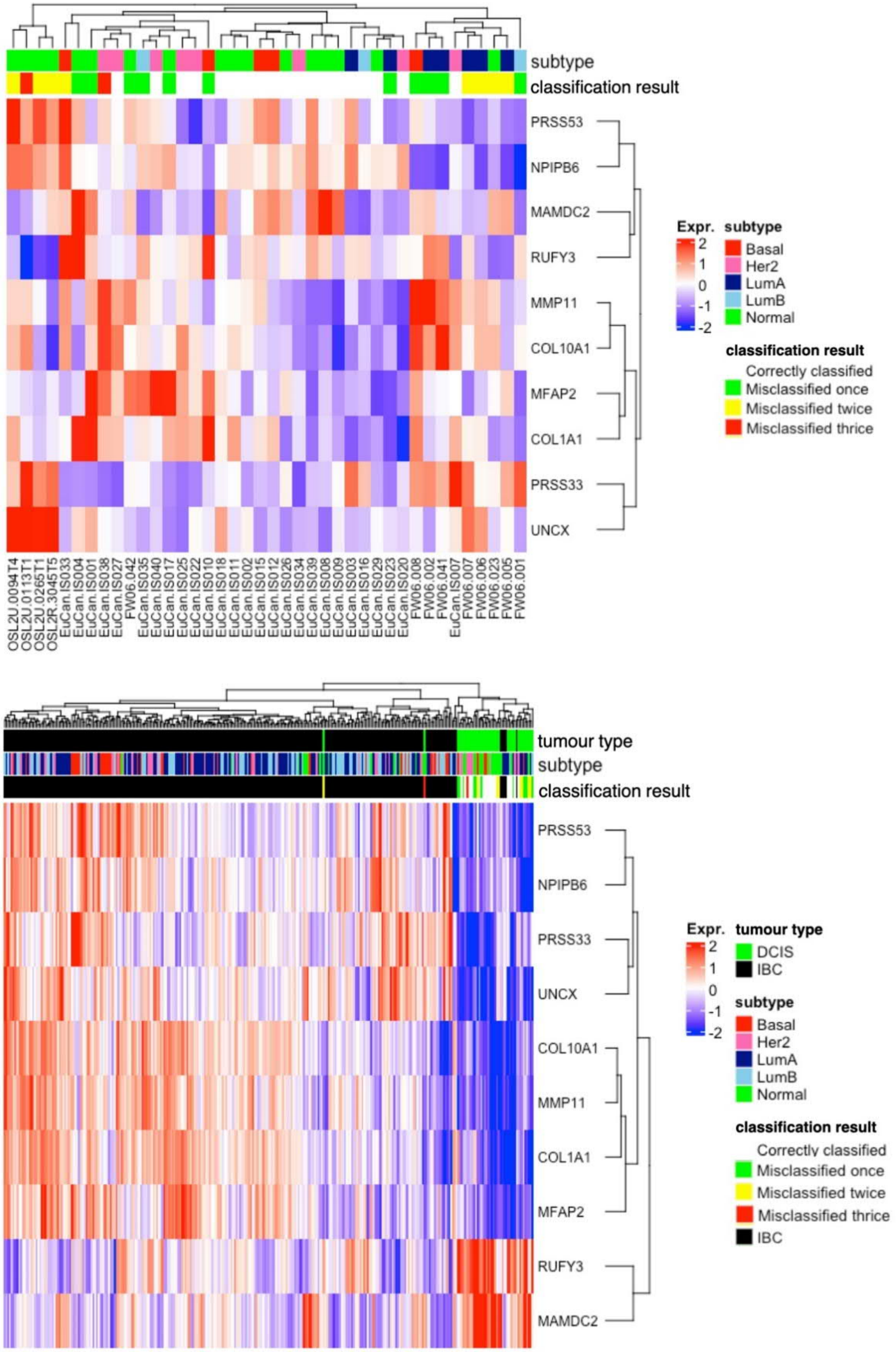

Supplementary Figure 7. Balanced accuracy of the 10-signature Lasso model at various classification cutoffs. The highest accuracy was achieved at cutoff of 0.03 (BA 0.93, sensitivity 1.00, specificity 0.85). The default cutoff of 0.5 led to rather poor accuracy (BA 0.61, sensitivity 0.22, specificity 1.00).

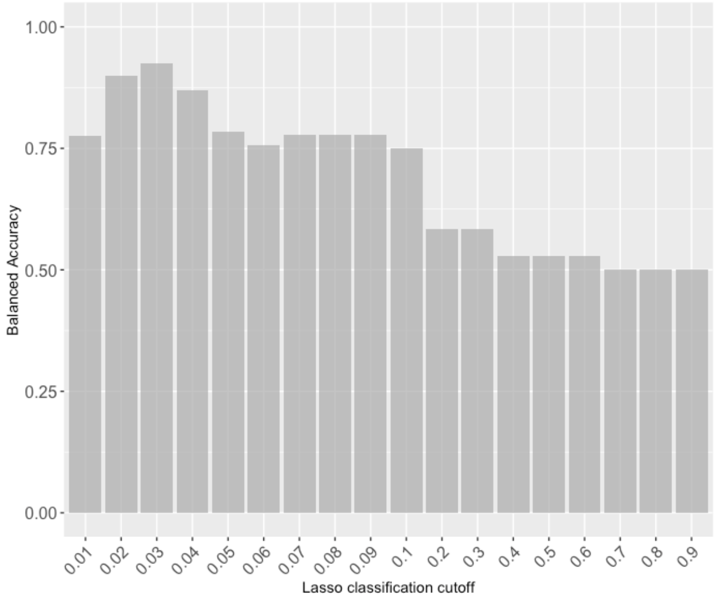

Supplementary Figure 8. Non-logged version of the class prediction probability in the validation cohort.

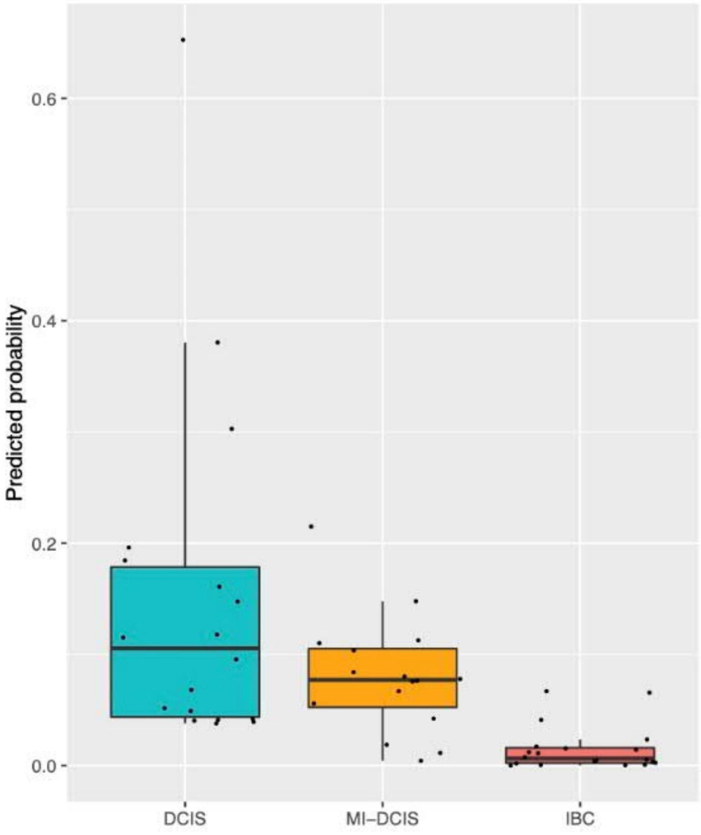

Supplementary Figure 9. Expression levels of the 10 genes in validation cohort after z-scoring.

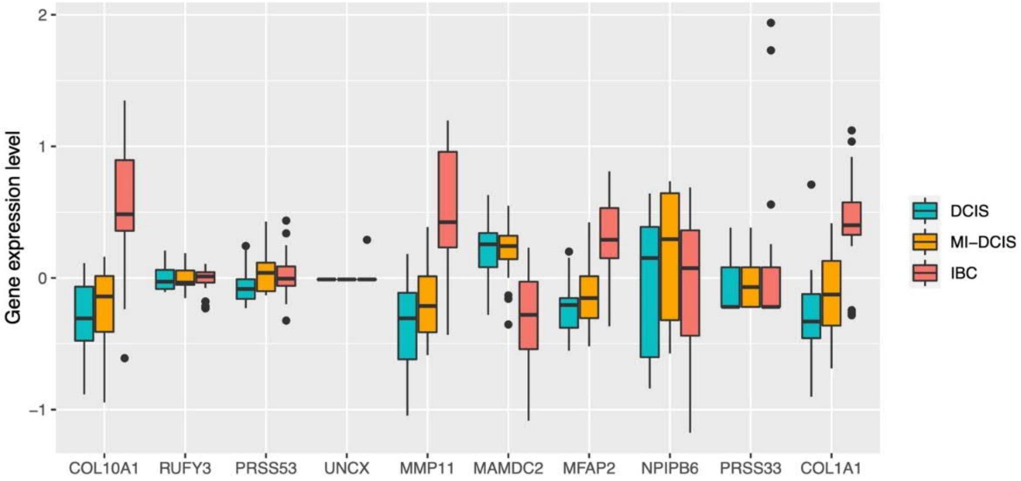

Supplementary Figure 10. Expression values of the 10 genes across the DCIS cases repeatedly misclassified as IBC after z-scoring.

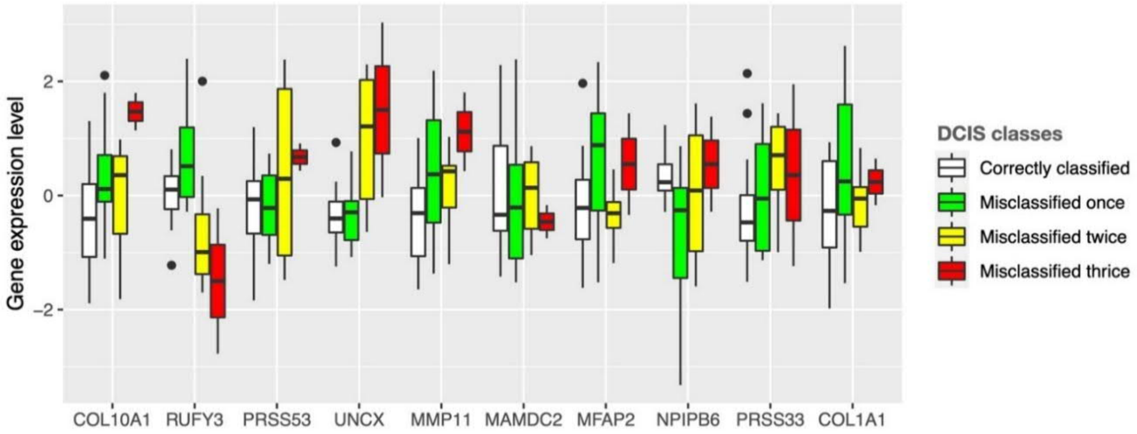

Supplementary Figure 11. Expression values of the 10 genes across the recurrence classes in the validation cohort after z-scoring.

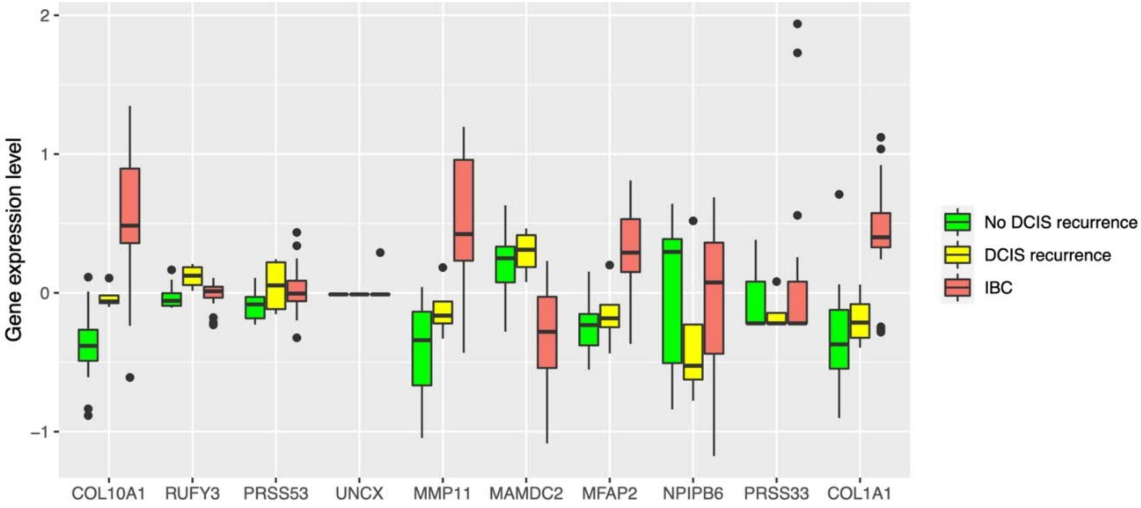

1.2 Supplementary Tables

Supplementary Table 1. Predictive contribution of omics profiles and their combinations to DCIS vs IBC classification using Lasso model. The data are shown as mean (standard deviation) over 10 CV folds in nested CV. Red values indicate the best result for each metric, and blue values close to the best or equal performance. Sensitivity, specificity and balanced accuracy are defined based on the default 0.5 probability cut-off of Lasso (see Methods). Individual ROC and PRCs of CV folds for the various omics data and their combinations are shown in Supplementary Figure 3.

	Sensitivity (DCIS)	Specificity (IBC)	Balanced accuracy	AU-ROC	AU-PRC	MSE	Number of features
**Expression**	0.827 (0.236)	0.996 (0.011)	0.910 (0.120)	0.992 (0.012)	0.972 (0.058)	0.132 (0.280)	20.9 (12.7)
**Methylation (gene-level)**	0.460 (0.304)	0.988 (0.012)	0.720 (0.116)	0.906 (0.077)	0.757 (0.152)	0.355 (0.400)	54.5 (15.1)
**Methylation (CpG-level)**	0.693 (0.255)	0.992 (0.024)	0.840 (0.069)	0.957 (0.032)	0.862 (0.122)	0.156 (0.313)	67.7 (7.7)
**Copy number**	0.512 (0.271)	0.973 (0.029)	0.740 (0.135)	0.887 (0.066)	0.617 (0.162)	0.204 (0.333)	158.3 (21.4)
**All (gene-level)**	0.863 (0.161)	0.996 (0.000)	0.930 (0.110)	0.995 (0.013)	0.973 (0.068)	0.0364 (0.080)	26.5 (13.2)
**All (CpG-level)**	0.822 (0.194)	0.996 (0.011)	0.910 (0.090)	0.995 (0.030)	0.979 (0.067)	0.0857 (0.237)	20.1 (10.3)

Supplementary Table 2. Predictive contribution of omics profiles and their combinations to DCIS vs IBC classification using Random Forest model.

	Sensitivity (DCIS)	Specificity (IBC)	Balanced accuracy	AU-ROC	AU-PRC
**Expression**	0.587 (0.299)	1.000 (0.000)	0.793 (0.149)	0.989 (0.014)	0.952 (0.060)
**Methylation (gene-level)**	0.290 (0.324)	0.992 (0.017)	0.641 (0.163)	0.889 (0.148)	0.728 (0.265)
**Methylation (CpG-level)**	0.213 (0.178)	0.996 (0.012)	0.605 (0.089)	0.799 (0.147)	0.550 (0.227)
**Copy number**	0.125 (0.137)	0.973 (0.011)	0.561 (0.067)	0.827 (0.149)	0.632 (0.296)
**All (gene-level)**	0.520 (0.254)	1.000 (0.000)	0.760 (0.127)	0.978 (0.047)	0.924 (0.157)
**All (CpG-level)**	0.473 (0.281)	1.000 (0.000)	0.736 (0.141)	0.987 (0.021)	0.956 (0.076)

Supplementary Table 3. Predictive contribution of omics profiles and their combinations to DCIS vs IBC classification using SVM model.

	Sensitivity (DCIS)	Specificity (IBC)	Balanced accuracy	AU-ROC	AU-PRC
**Expression**	0.568 (0.279)	1.000 (0.000)	0.784 (0.139)	0.974 (0.065)	0.817 (0.356)
**Methylation (gene-level)**	0.083 (0.136)	0.996 (0.012)	0.540 (0.062)	0.920 (0.099)	0.695 (0.269)
**Methylation (CpG-level)**	0.095 (0.255)	1.000 (0.000)	0.548 (0.069)	0.939 (0.060)	0.771 (0.181)
**Copy number**	0.000 (0.000)	1.000 (0.000)	0.500 (0.000)	0.834 (0.103)	0.627 (0.234)
**All (gene-level)**	0.397 (0.254)	1.000 (0.000)	0.698 (0.127)	0.982 (0.036)	0.839 (0.299)
**All (CpG-level)**	0.387 (0.261)	1.000 (0.000)	0.693 (0.131)	0.976 (0.022)	0.803 (0.267)

Supplementary Table 4. Effect of pseudo labeling on the accuracy of the Lasso model.

	Expression (original label)	Expression (pseudo label)	Methylation (original label)	Methylation (pseudo label)
**AU-ROC**	0.996 (0.012)	0.994 (0.023)	0.963 (0.032)	0.962 (0.070)
**AU-PRC**	0.982 (0.058)	0.983 (0.055)	0.844 (0.122)	0.906 (0.105)
**Number of misclassified samples**	7	1	10	6

Supplementary Table 5. Classification accuracy of 10-gene Lasso model and other risk scores.

	Sensitivity (DCIS)	Specificity (IBC)	Balanced accuracy	AU-ROC	AU-PRC
**10-gene Lasso score**	0.897 (0.137)	0.985 (0.026)	0.941 (0.071)	0.992 (0.014)	0.972 (0.073)
**Invasiveness score**	0.000 (0.000)	1.000 (0.000)	0.500 (0.000)	0.842 (0.078)	0.473 (0.154)
**ROR score**	0.000 (0.000)	1.000 (0.000)	0.500 (0.000)	0.601 (0.104)	0.179 (0.055)
**Oncotype DX® DCIS Score**	1.000 (0.000)	0.000 (0.000)	0.500 (0.000)	0.550 (0.096)	0.120 (0.033)
